# Needs assessment to strengthen capacity in water and sanitation research in Africa: experiences of the African SNOWS consortium

**DOI:** 10.1186/1478-4505-12-68

**Published:** 2014-12-15

**Authors:** Paul R Hunter, Samira H Abdelrahman, Prince Antwi-Agyei, Esi Awuah, Sandy Cairncross, Eileen Chappell, Anders Dalsgaard, Jeroen HJ Ensink, Natasha Potgieter, Ingrid Mokgobu, Edward W Muchiri, Edgar Mulogo, Mike van der Es, Samuel N Odai

**Affiliations:** The Norwich Medical School, University of East Anglia, Norwich, NR 4 7TJ UK; Department of Environmental Health, Faculty of Science, Tshwane University of Technology, Private Bag X680, Pretoria, 0001 South Africa; Department of Family & Community Medicine, University of Gezira, PO Box 20, Wad Medani, Sudan; Civil Engineering Department, Kwame Nkrumah University of Science & Technology, Kumasi, Ghana; Environmental Health Group, Faculty of Infectious and Tropical Diseases, London School of Hygiene and Tropical Medicine, Keppel Street, London, WC1E 7HT UK; Department of Veterinary Disease Biology, Faculty of Health and Medical Sciences, University of Copenhagen, Grønnegårdsvej 15, DK-1870 Frederiksberg C, Denmark; Department of Microbiology, University of Venda, P/Bag X5050, Thohoyandou, South Africa; Faculty of Engineering, Egerton University, PO Box 536, Egerton, Njoro, Kenya; Department of Community Health, Mbarara University of Science and Technology, PO BOX 1410, Mbarara, Uganda

**Keywords:** Research, Water, Sanitation, Training, Water

## Abstract

**Background:**

Despite its contribution to global disease burden, diarrhoeal disease is still a relatively neglected area for research funding, especially in low-income country settings. The SNOWS consortium (Scientists Networked for Outcomes from Water and Sanitation) is funded by the Wellcome Trust under an initiative to build the necessary research skills in Africa. This paper focuses on the research training needs of the consortium as identified during the first three years of the project.

**Methods:**

We reviewed the reports of two needs assessments. The first was a detailed needs assessment led by one northern partner, with follow-up visits which included reciprocal representation from the African universities. The second assessment, led by another northern partner, focused primarily on training needs. The reports from both needs assessments were read and stated needs were extracted and summarised.

**Results:**

Key common issues identified in both assessments were supervisory skills, applications for external research funding, research management, and writing for publication in the peer-reviewed scientific literature. The bureaucratisation of university processes and inconsistencies through administration processes also caused problems. The lack of specialist laboratory equipment presented difficulties, particularly of inaccessibility through a lack of skilled staff for operation and maintenance, and of a budget provision for repairs and running costs. The lack of taught PhD modules and of research training methods also caused problems. Institutionally, there were often no mechanisms for identifying funding opportunities. On the other hand, grantees were often unable to understand or comply with the funders’ financial and reporting requirements and were not supported by their institution. Skills in staff recruitment, retention, and performance were poor, as were performance in proposal and paper writing. The requirements for ethical clearance were often not known and governance issues not understood, particularly those required by funders.

**Conclusions:**

SNOWS believes that working with African universities to develop networks that support African-led research driven by the local context is an effective approach to develop and retain research skills needed to change policy and practice in water, sanitation, and hygiene in Africa.

**Electronic supplementary material:**

The online version of this article (doi:10.1186/1478-4505-12-68) contains supplementary material, which is available to authorized users.

## Background

Inadequate access to safe water and improved sanitation is one of the most important preventable causes of disease burden in children living in low-income countries [1, 2]. Although access to safe water and improved sanitation has improved significantly for many countries in recent years, some authors have expressed doubt over claims that the Millennium Development Goal on access to safe drinking water has been met [3]. In any event, sub-Saharan Africa still lags behind the rest of the world on access to both drinking water and sanitation [4]. Although many financial resources have been directed at improving the situation, this has not always resulted in an increased uptake or use of facilities, let alone improvements to public health. An example of this is India’s Total Sanitation Campaign, which saw over 90 million latrines built since 1992, but uptake and use of the built facilities is still far from 100%, with, in some instances, more than 50% of intended users still practicing open defecation [5]. There is, therefore, a strong and clear need for good quality research into the most effective approaches for reducing the disease burden in Africa from unsafe water and inadequate sanitation.

Whilst there has been research on water and sanitation in Africa over recent decades, the topic still remains a relatively neglected one in comparison to other research topics [6]. Diarrhoeal disease, a major outcome of inadequate access to water and sanitation, is recognised as being a relatively neglected area for research funding related to its disease burden compared to other, more “fashionable”, topics such as malaria and HIV/AIDS. In a study of the uptake of research into policy in low- and middle-income countries, Hennink and Stephenson noted several barriers to uptake that included lack of appropriate packaging of research findings and the tendency for findings to be disseminated only in academic circles [7]. To overcome these barriers, having research in Africa led by African institutions is a first but powerful step. African-based research groups would be better able to identify areas and topics for research that fulfil the needs of local communities through community engagement research and social activities.

A strong African-based research effort would also provide the environment to nurture the experts and leaders of tomorrow. Such research effort would also give these leaders of tomorrow the skills to lead much needed improvements regarding water, sanitation, and hygiene issues, which will induce real improvements in public health. This should not be restricted to mean that developing research skills and capacity is of value just for career researchers; research skills are needed by a broader range of professionals. Such research skills are of value for service evaluation or planning in many professional spheres and skills in operational research should be part of all professional training courses from undergraduate level upwards. In practice, university is the best place for them to learn those skills, and their teachers will teach them better if they also have experience of applying them. Research, then, is not just a means to advance the professions in the Water, Sanitation, and Hygiene (WASH) sector, it is also a fundamental part of practising those professions in low-income communities.

It is in the above context that the Scientists Networked for Outcomes from Water and Sanitation (SNOWS) consortium came together in response to a call from the Wellcome Trust African Initiative to help build capacity for environmental health research, with a particular focus on water and sanitation and its associated impact on public health in Africa. This paper describes the first three years of the consortium and reports the outcome of a needs assessment undertaken regarding the southern partner universities’ research infrastructure and other capacity building needs. Thus, the objective was to identify and discuss the main challenges faced by the southern SNOWS partners in establishing capacity to conduct interdisciplinary research within water and sanitation.

### The consortium

The aim of the consortium, with the help of the Wellcome Trust is “*to build African capacity for interdisciplinary research in water supply, sanitation, and environmental health, bringing together universities from across the continent, with research-active universities in the North*”. The consortium consisted of six African and three European universities. The six African universities were the Kwame Nkrumah University of Science & Technology, Ghana (also hosted the consortium director); Mbarara University of Science and Technology, Uganda; University of Gezira, Sudan; Egerton University, Kenya; Tshwane University of Technology, South Africa; and University of Venda, South Africa. The three European universities were the London School of Hygiene & Tropical Medicine, UK (also hosted the deputy director); University of East Anglia, UK; and University of Copenhagen, Denmark. One of the key strengths of the consortium is the multidisciplinary and interdisciplinary nature of the key individuals leading their institution’s involvement. Within the consortium are found microbiologists, epidemiologists, public health specialists, anthropologists, social scientists, engineers, and administrators. Whilst all of the African universities aspire to being research-active institutions, they are at very different stages in their organizational development in this regard. Some of the partners had a pre-existing active research programme whilst others had minimal prior involvement in research. With the single exception of South Africa, the African region and the water and sanitation sector have both tended to be low priorities in the allocation of research funding. The chronic shortage of research funding and the heavy workload of teaching and administration on many academic staff, are the most likely reasons for the difficulties that many colleagues in the African partners have in being able to pursue research careers. However, this was nothing which could not be overcome by a participatory approach and, as we point out below, it was not unknown in Northern universities too.

## Methods

One of the earliest activities of the consortium, and a requirement of the Wellcome Trust, was to undertake a needs assessment for research capacity building in the African partner universities. After an initial, unsuccessful attempt to collect information by e-mailed questionnaire, the consortium conducted two independent needs assessments in a more proactive manner. The first was led by the University of Copenhagen, and was based around a visit of two consultants from Copenhagen supported by a third member from one of the other African institutions. Each African university was visited and, prior to the visit, key staff were asked to complete a detailed needs mapping questionnaire, though, like the emailed questionnaire, this was not well completed by most universities and was not subsequently analysed for this paper. During each visit, discussions were held with groups of key stakeholders, representing senior management, academic staff (at any grade provided they had an interest in WASH research), administrative staff, PhD students, and Masters students. After these visits, the consultancy team submitted a report to the host institutions which consisted of a range of recommendations for strengthening the research capacity of the institution, jointly arrived at by the consultants and the relevant southern partner. This needs assessment focused primarily on the management and structural needs within the southern universities. A report was produced for each southern partner and it was these reports that were analysed for this paper. The questionnaires used in this process are included as Additional file 1.

In addition, a training needs assessment using an in-depth semi-structured interview with a key informant from each African university was undertaken by a staff member from the University of East Anglia. This was undertaken before the management needs assessment was completed. The key informant was the senior academic leading each university’s involvement in SNOWS. The views of the key informant were subsequently verified against the views of other staff from the same university. This needs assessment culminated in a single report for the entire consortium.

This paper is based on a review of the training needs assessment and the reports from the Copenhagen team. When identifying key issues the focus was on those issues that were common to most partners, especially those factors identified in both the Copenhagen assessment and the training needs assessment. One researcher read through all reports and highlighted statements relating specifically to expressed needs, those needs that were expressed in three or more of the reports were then extracted for comment. These statements were then clustered into major themes. A second researcher then reread the reports and checked for completion. The extracted statements were also discussed in a round table meeting with all African partners to check for completeness and finally circulated to all southern partners for comment along with the first draft of this paper.

## Results

The three main areas that required focus and strengthening that came out of the needs assessment process were the management of MSc and PhD research programmes (in particular research student supervision), the application for, and management of external research funds, and writing for publication in peer-reviewed journals. Short summaries of the main issues identified are listed in Tables 1 and 2.

**Table 1:** **Summaries of key points in regard to research student supervision**

Point number	Summary
1.1	Poor structure, funding, and organisation lead to a low completion rate, a high drop-out rate, and students taking on average 3 years to complete a Masters and to 6 years to complete a PhD.
1.2	Research supervision is under-resourced, poorly organised, and lacks any appropriate reward structure, or quality control, so staff may give little practical guidance to many students.
1.3	Many faculty staff may attempt to supervise 5 to 50 research students alongside their teaching and research duties, an unsustainable workload.
1.4	Staff may lack the communication skills used to guide research students, reverting to the lecture-type of approach used on undergraduates, which fails to develop relevant problem-solving skills. For example, some faculty have no email address, which frustrates student communication.
1.5	Research students find it very hard to regularly meet their supervisor or co-supervisor even separately, and may receive conflicting advice from both.
1.6	University regulations on graduate degrees are often poorly communicated, out-of-date, and not well understood by faculty or students, further complicating the smooth running of the programme and again contributing to delays.
1.7	Research students are normally allowed to choose a research topic that interests them (with no guidance provided) as was common in Europe in the 1980s. This creates four issues, namely an inability to find a competent supervisor for that topic, a difficulty in demonstrating the necessary originality and research skills in the degree, a lack of funds or funding potential, and more dropouts.
1.8	Inadequate laboratory facilities in terms of space, access to supplies and chemicals needed, specialised laboratory equipment and the expertise to use it, maintain it, or repair it. Some students are encouraged to switch to a theoretical degree to avoid the delays around effective lab facilities access.
1.9	Many students felt that specialised equipment should not be bought unless paid technicians could be provided who would explain how to use it, maintain it properly, and thus improve its value and life expectancy. Expensive items had been broken or damaged through lack of laboratory technicians.
1.10	If there were competent lab technicians they could provide useful hands-on training to research students in how to use the relevant laboratory equipment.
1.11	Most of the research students lacked a good grounding in research methods at both Masters and PhD level, which could be taught as a common course at different levels. This contributed to more delays and research mistakes.
1.12	Some research students found good teaching in these research methods in other universities or faculties, which they then had to pay for separately, creating more delays. A system of credits should be set-up enabling students to get good relevant fill-in teaching in other centres without being financially penalised as they are already paying research degree course fees.
1.13	Most universities lack an organised office that attempts to find and match funding for research degrees with the sources of funds available nationally or internationally. This information is lacking or not regularly shared.
1.14	Reliable internet access is often lacking for research students. When available, students tend to search on Google.com instead of the science research databases such as Google Scholar, PubMed, etc.
1.15	Some research degrees require publication of one or two peer-reviewed scientific articles, and provide no help achieving this, creating another stumbling block leading to incomplete degrees. Very few students may get a teaching post which often enables them to finally get over this obstacle.

**Table 2:** **Summaries of key points in regard to externally funded research**

Point number	Summary
2.1	Most partners said that there was no centralised information on calls for research bids and no organised sharing of information to improve the university’s record bidding for research projects.
2.2	While the universities had a few staff successful in obtaining external funding, there seemed to be no clear strategy regarding research, little leadership or guidance from the senior staff, and also no training to share the existing expertise or “research strategy”, if it existed. Nor are there succession plans for research leadership.
2.3	Local university guidance on appropriate terms in new research contracts would be helpful, too often new researchers accept contract clauses that are impossible to keep to.
2.4	Guidance from senior researchers and administrators should give the lead researcher a good idea of local university rules on finance, disbursements, and end-of-year carryovers. Reporting and procurement are likely to clash with donor requirements and therefore need early resolution.
2.5	A clear plan as to who will handle the research project finance and with what type of account would minimise the common delays to disbursements. Authorising back-up staff signatories for payments and reports helps the researcher to complete tasks, reports, and procurement on time.
2.6	Often, researchers new to outside funding are unsure of how to manage the recruiting, contracting, and management of temporary, fixed-term, and community-based staff or volunteers, which can lead to problems as these individuals may become responsible for doing important work in very isolated areas with poor communications and access. Cash payments might be necessary.
2.7	Researchers in these universities too often underestimated the time needed to prepare for and gain ethics approval for the research proposed and many staff feel that the committee may at times lack the knowledge or training needed to properly protect the interests of the public.
2.8	Another common weak point for researchers was their lack of access to secure and back-up university servers to store the research data on. At times that caused certain donors to withdraw research grants. Planning to protect samples from fire, flood, theft, or copying can also be a challenge in isolated rural areas.
2.9	In terms of research ethics, challenges can include scanty knowledge of the research protocol and a reluctance to inform the ethics committee of changes to that protocol. Likewise, a reluctance to inform the authorities of any shortcomings in terms of confidentiality or re-usage of medical samples collected previously for different purposes is not that unusual.

### Research student supervision

Probably, the key issue hampering growth in research capacity is the difficulty in supervision of postgraduate research students and young researchers (Table 1, points 1.2, 1.3, 1.4, and 1.5). It is generally felt that research students, at both Masters and Doctoral level, should be supervised by researchers who have themselves obtained a PhD degree. Recruiting students onto such programmes does not seem to be a problem, however, most of the African universities simply do not have enough sufficiently qualified researchers to provide a robust supervisory environment. This problem is reflected in the observation that many academics reported having unrealistically high numbers of students to supervise. This, in turn, led many students to complain that it was difficult to get time with their supervisors.

In order to address this weakness, all of the African universities were giving priority to enable their own lecturers to work for a PhD. Unfortunately, it was often the case that once the lecturers had obtained their PhDs, they moved on to new jobs. This was a particular problem for the rural universities who reported that lecturers who obtained their PhDs in post, would usually move to universities in their capital cities. The perception was that jobs in the capital were better paid and offered better career progression opportunities, resulting in a better quality of life.

A number of the other key points related to the quality of supervision and a lack of understanding of the requirements of the role (points 1.4, 1.6, and 1.7). A common complaint was that students and supervisors were often unclear about the university’s regulations with regard to research degrees.

It is clear from the needs assessments, and from talking to African colleagues and students, that a high proportion of time is spent in navigating university rules and regulations. Whilst this may also happen to some extent in the European partner institutions, the additional challenge appears to come either from a lack of communication and, therefore, a lack of clarity in governance issues, or from administration that does not pay enough attention to explaining the rules and regulations to research staff. There is also a reluctance to supervise PhD students, declared by half of the African partners in the first needs assessment, because of the time it takes away from their commitments to teaching and other university duties. The challenges faced in this one area alone produce bottlenecks to progress that are frustrating for all concerned. In this context, the desire for ‘incentives’ to encourage supervision can be understood (as indicated in three of the six universities); however, the longer term need for university staff to conduct research requires a more integrated support (points 1.2, 1.3, 1.6, 1.8, and 1.9).

Most of the remaining key points in this section relate to access to research resources, especially equipment (points 1.8, 1.9, 1.10, 1.11, and 1.14), and training in research methods (points 1.11, 1.12, and 1.13). The point is well made that, for many of the African universities, access to laboratory equipment is often problematic, and even when equipment has been obtained there is often a lack of skilled technicians able to support the use of such machines. Where specialised equipment had been purchased, maintenance and repair costs were often not included in faculty, research, or teaching budgets and, as a result, the value and functioning life were considerably reduced (point 1.9). Students were sometimes encouraged to switch to a theoretical research project to avoid pressures on equipment and additional running costs (point 1.8). Clearly, expenditure on research equipment that will not be used either because of inappropriate choice of equipment, or because of the lack of people able to run this equipment is a waste of resources.

The issue of lack of training in research methods is also particularly relevant. Unlike the case in European universities, few of the PhD courses from the African universities had any taught modules on quantitative and qualitative research methodologies. Consequently, it was difficult for students to decide on the most appropriate research methods to use for their research. This was compounded by the fact that, because the supervisors were expected to supervise a very wide range of projects, they were often not expert in the appropriate research methodologies, though in some universities the requirement for multiple supervisors for each PhD student went some way to obviate this.

### Externally funded research

Many of the African universities were poorly organised in being able to identify and attract external research funding. Whilst there were examples of success from all the African universities, most said that there was no centralised strategy to help academics identify funding opportunities and apply for funds (Table 2, points 2.1, 2.2, and 2.3). When researchers had been successful at winning funds, they often did not understand the university’s rules on research grant management and administrators often found it difficult to understand and comply with donors’ requirements (points 2.4 and 2.5). At times, local university policies were reported as actively conflicting with donor’s requirements. These issues are illustrated by the following quotes from key informants:

“*The rules in terms of research staff recruitment, finance, payments, and reporting are not very clear and often not well understood by graduate students helping with research. You end up having to do a lot of things yourself, especially things involving other departments like finance, procurement and audit*.”

“*We try to do good research but the university systems like payments, audit, and finance, which regulate how we can use research funds are put in place to manage the teaching process – they often don’t suit research work at all. Some changes would help us do research well*.”

This issue was illustrated very clearly in the management of the SNOWS grant where several of the African partners struggled in the early stages to understand the financial reporting and governance requirements of the Wellcome Trust. Indeed, it is true to say that so much effort was put into this aspect of the grant in the early years that the actual scientific work suffered as a result. A particular issue (point 2.6) was the need for a cash economy when paying staff in isolated areas with poor communications.

In the case where researchers were new at managing externally funded research projects, they often felt they lacked the management skills for recruiting and retaining their contract researchers and getting the best work from them (point 2.6). Another particularly problematic management issue was contact with the review process for those research projects that needed ethical clearance (points 2.7 and 2.9). Often staff and students had little understanding of the need for ethical review, what sort of projects need ethical clearance, and when are ethical committees needed to be informed of changes in protocol, etc. This was to a certain extent compounded in those universities where ethical review committees were a relatively recent innovation, and where the committee members themselves had little experience.

### Writing for publication

Many of the researchers and students expressed concerns about their ability to write papers in English of a standard suitable for publication in the international peer review literature. The move to open access publication with the associated costs to the authors was also a concern with African researchers expressing concern over their ability to afford these fees.

## Discussion

This paper reports the findings from the needs assessments conducted in African partner institutions of the SNOWS consortium, a consortium funded by the Wellcome Trust with the intention of building the capacity of African universities to do research in environmental health and, in particular, in water and sanitation. A number of themes were identified, broadly classified into research student supervision, management of external research grants, and writing for publication. Probably the most difficult issue was the current lack of suitably qualified research supervisors in many of the universities, but this was compounded by unclear rules and policies, a lack of equipment, maintenance of equipment, absence of good technicians to look after the equipment, and a lack of taught courses in research methodology. For externally funded research, many of the African partner universities at the time of the needs assessment found it difficult to support their academics, to identify funding opportunities, and to apply for grants. Where academics had been successful at winning external funding, many struggled to understand their university’s or their funder’s financial rules and policies. Further, uncertainty existed with regard to ethical review. In many ways, the issues identified in this work are similar to what was found in the late 1990s prior to substantial funding of the Gates Malaria Partnership [8]. The findings are also consistent with other reports on research supervision from Africa [9–11].

It would be wrong to think that all of the issues identified in this paper are unique to African universities, or indeed even to universities in low- and middle-income countries. Examples of problems and conflicts in the supervisory process for higher degrees are frequent findings across the globe [12–15]. A key aspect of the African experience which is probably not as severe in the European context is the much poorer staff to student ratio exacerbated by the pressure to take on students whose research interests may not coincide with those of the supervisor. Many of the African partners also expressed their concern over their supervisory skills. Whilst the latter has been addressed in part by training and mentoring of supervisors, much still needs to be done for research supervision excellence to be mainstreamed into African universities. It is suggested that only when long term funding of such research is available, will the skills and expertise of the research mentors and supervisors become fixed. A good example of the success of this type of approach comes from the Gates Malaria Partnership grant [8].

Similarly, many of the institutional hurdles to good research and quality research supervision are not unique to the African setting. Several authors from Europe and North America have commented on the increasing bureaucratisation of universities or research institutes in the developed North [16–18]. Evidence for this comes from findings that, whilst research and teaching staff numbers have remained similar or even declined over the past decades, administration staff numbers have increased dramatically [16]. In Europe, many academics now cite over-burdensome university bureaucracy limiting their ability to do good research, describing it as more problematic than difficulties in finding funds [16]. There is indeed some evidence to support the hypothesis that research output is inversely proportional to the level of bureaucracy in the organisation [16–18]. Whilst there was evidence of creeping bureaucratisation in the African context, probably a bigger problem was a lack of organisational knowledge on how to support academics to obtain research funding, and how to manage these grants effectively once obtained. By contrast, in the report on research capacity building in Africa written specifically about the Wellcome Trust’s African Institutions Initiative [19], the evaluators found that “*The degree to which guidelines exist on specific administrative processes varies widely across the region, and many aspects of research administration are implemented through improvisation and without consistent adherence*”. Nevertheless, it could certainly be argued that academic bureaucratisation is a problem that threatens to become endemic in Africa and, if so, could impair further growth in research capacity.

However, there is an opportunity to learn from our African and European universities on this point. The first of the two quotes in the section on external funding highlights the need for greater understanding of the governance systems in African universities. This provides an opportunity for the creation of a governance system that satisfies international best practice within a local context, without emulating the administrative-heavy bureaucracies that are burgeoning elsewhere. Our findings in the needs assessments can be used to understand what each African institution needs to function effectively and sustainably while at the same time meeting the requirements of donor agencies.

Another point that was highlighted in this assessment was the issue of ethical review. Relative to the developed world, ethical review committees are a recent innovation in Africa [20, 21]. As recently as 2004, a study found that 44% of research projects in the developing world had been reviewed only in the funding country [22]. Perhaps reflecting this more recent development was a limited knowledge of how ethics committees operate, and what issues need to be brought to their attention as, for example, when changes are made to a study protocol. Such confusion is perhaps understandable as many research projects undertaken in Africa will have ethical review in the funding country as well as the African country, and the opinions of the different committees do vary. Indeed, where joint review is undertaken, there is often disagreement between committees [23–25]. Such disagreements between countries only add to confusion with the process.

A strong message from both of our needs assessments is that the issues of financial and research management and governance are inextricably linked to the success of the African institutions. The responses of all six partners reflected their perception that, unless their university provided them, as students, teachers, and researchers, with appropriate and effective support, they could not do their jobs and would not be able to achieve their personal aims nor those of their institution. In more specific terms, a number of issues identified by our analyses probably require a more generic shift in the way funds for research in Africa are administered. It is vital that good financial governance has to be maintained in order to ensure that research funds end up being spent on the research for which they were awarded. However, there has to be a more flexible and supportive approach from funders, especially for work done in universities outside of the mainstream.

Many of the challenges in building a sustainable capacity strengthening programme are experienced by each of the seven consortia in the Wellcome Trust’s African Institutions Initiative programme [19]. SNOWS developed its own strategic plan in response to the needs assessments and, in so doing, adopted a number of capacity building models. One was networking, which had shown up in both needs assessments and was of relevance to both research and support staff. The network model has been shown to be particularly effective in producing sustainability after training [26], an area in which many other models struggle, and has proven effective in combination with other strategies, such as mentoring, among project support staff.

## Conclusions

In conclusion, we believe that working alongside African universities as they develop and support their own research programmes aimed at addressing problems that they have identified, is likely to be one of the most effective approaches to strengthening research capacity on the continent. Such an approach is more likely to generate changes in policy and practice that make real and sustained improvements to the plight of the people in Africa with regards to water, sanitation, and hygiene issues. We further believe that, for problems primarily affecting rural communities, research is best done by universities with a focus on work in rural environments. It is these universities that often face the most difficulty in sustaining their research programmes. To improve the amount and impact of the research from these universities requires training and resources. It also, in our view, requires a reappraisal from funders on how they support and manage awards to such universities. This improved tailored support would include the development of funding models that provide long term commitments and support to enable them to develop their research skills.

## Electronic supplementary material

Additional file 1: **Zip file containing questionnaires used in the needs assessment.** (ZIP 338 KB)

## Abbreviations

SNOWS: Scientists Networked for Outcomes from Water and Sanitation
WASH: Water, Sanitation, and Hygiene.
