# Supplementary material for: Needs assessment to strengthen capacity in water and sanitation research in Africa: experiences of the African SNOWS consortium
Source: Health Res Policy Syst. 2014 Dec 15;12:68. doi: 10.1186/1478-4505-12-68 (PMC4274706; doi:10.1186/1478-4505-12-68)
Supplement: Supplementary file 1 — Additional file 1: Zip file containing questionnaires used in the needs assessment. (ZIP 338 KB) [file 12961_2014_366_MOESM1_ESM.zip › SNOWS - Semi structured questionnaire - Master students (1).docx]

SNOWS – Questionnaire for: *Current or recently graduated post-graduate (Master-level) students*

*Please, circle the correct code or fill in the response*

| **A. BACKGROUND AND PERSONAL INFORMATION** | | | | | |
| --- | --- | --- | --- | --- | --- |
| 1 | Date of filling the questionnaire (dd/mm/yyyy): | | | | |
| 2 | At which of the following universities did you register for your post-graduate study?   1. Egerton University, Kenya 2. Kwame Nkrumah University of Science & Technology, Ghana 3. Mbara University of Science & Technology, Uganda 4. Tshwane University of Technology, South Africa 5. University of Gezira, Sudan 6. University of Venda, South Africa | | | | |
| 3 | Category of respondent:   1. Currently registered as a post-graduate student; specify Master type/subject:………………. 2. Recently graduated post-graduate student (within the last two years); specify Master type/subject:……… | | | | |
| 4 | Sex:   - - - 1. Male       2. Female | | | | |
| 5 | Age (in years): | | | | |
| 6 | Highest degree attained (below Master-level):   1. Bachelor degree; specify subject:……………………….. 2. Other; specify:………………………….. | | | | |
| 7 | Awarding university (of highest degree attained below Master-level); specify name and country:…………. | | | | |
| 8 | Year of graduation (of highest degree attained below Master-level): | | | | |
| **B. REGISTRATION AND FUNDING OF POST-GRADUATE TRAINING** | | | | | |
| 9 | Department of registration of current or recently completed postgraduate study: | | | | |
| 10 | Date of official registration as post-graduate student (month and year): | | | | |
| 11 | (Expected) date of submission of Master-level thesis (month and year): | | | | |
| 12 | (Expected) date of graduation (month and year): | | | | |
| 13 | Where does/did funding for your post-graduate study come from?   1. This university 2. A national funding agency 3. An international university 4. An international funding agency 5. Other; specify which one(s):…………………… 6. I don’t know | | | | |
| 14 | Does/did the post-graduate budget make provisions for you to travel internationally for meetings or conferences?   1. Yes 2. No 3. I don’t know | | | | |
| 15 | Does/did the post-graduate budget make provisions for you to travel internationally for course participation?   1. Yes 2. No 3. I don’t know | | | | |
| 16 | Did you travel internationally in connection with your post-graduate work?   1. Yes; specify where (country) and why (purpose):…………. 2. No | | | | |
| 17 | Do/did you know the total amount of your post-graduate budget?   1. Yes; what is/was the approximate amount (specify currency)?................... 2. No | | | | |
| **C. RULES AND REGULATIONS FOR POST-GRADUATE TRAINING PROGRAMMES** | | | | | |
| The following questions refer to the university where you registered for your post-graduate study | | | | | |
| 18 | Has the university developed any written rules and regulations governing their post-graduate programmes?   1. Yes; specify what has been developed:………………………. 2. No [go to 20] 3. I don’t know [go to 20] | | | | |
| 19 | Do you have a copy, hard or electronic, of any of these rules and regulations?   1. Yes; specify which documents you have:………………………… 2. No | | | | |
| 20 | How well do you feel that you know the rules and regulations governing the post-graduate programmes at the university?   1. Very well 2. Well 3. Poorly 4. Very poorly [go to 22] | | | | |
| 21 | What are the sources of your knowledge about the rules and regulations? | | | | |
|  | 1 | University or faculty handbook/guide | | 1. Yes 2. No | |
|  | 2 | Departmental handbook/guide | | 1. Yes 2. No | |
|  | 3 | University website | | 1. Yes 2. No | |
|  | 4 | Official briefing | | 1. Yes 2. No | |
|  | 5 | Informal discussions with university staff | | 1. Yes 2. No | |
|  | 6 | Informal discussions with fellow students | | 1. Yes 2. No | |
|  | 7 | Other; specify:……………………………. | | 1. Yes 2. No | |
| 22 | How accessible are the rules and regulations?   1. Highly accessible [go to 25] 2. Moderately accessible [go to 24] 3. Difficult to access 4. I don’t know [go to 25] | | | | |
| 23 | Please explain in which way access is difficult:………………….. | | | | |
| 24 | Do you have any suggestions for making the rules and regulations more accessible to those who need them?   1. Yes; specify how:………………………… 2. No | | | | |
|  |  |  |  |  |  |
|  |  |  |  |  |  |
|  |  |  |  |  |  |
| 25 | How do find the relevance of the rules and regulations to post-graduate students   1. Highly relevant 2. Moderately relevant 3. Irrelevant; specify why:……………… 4. I don’t know | | | | |
| 26 | How do find the usefulness of the rules and regulations to post-graduate students   1. Highly useful 2. Moderately useful 3. Useless; specify why:……………… 4. I don’t know | | | | |
| 27 | How do find the reasonableness of the rules and regulations to post-graduate students   1. Highly reasonable 2. Moderately reasonable 3. Unreasonable; specify why:…………….. 4. I don’t know | | | | |
| 28 | What could be done to make the rules and regulations more relevant, useful and/or reasonable? | | | | |
| 29 | To what extent have you personally used or referred to the rules and regulations?   1. Very frequently 2. Occasionally 3. Rarely; specify why:……………......... 4. Never; specify why:………………… | | | | |
| **D. ADMINISTRATION AND ORGANISATION OF POST-GRADUATE PROGRAMMES** | | | | | |
| The following questions are addressing the administrative mechanisms for post-graduate programmes at this university. The responsible body within the university structure is referred to as the “Post-Graduate Administration”. | | | | | |
| 30 | How do you find the responsiveness of the Post-Graduate Administration towards meeting the needs of post-graduate students?   1. Highly responsive 2. Moderately responsive 3. Poorly responsive; specify why:…………….. 4. I don’t know | | | | |
| 31 | How do you find the effectiveness of the Post-Graduate Administration in delivering services to post-graduate students?   1. Very effective 2. Moderately effective 3. Ineffective; specify why:…………….. 4. I don’t know | | | | |
| 32 | How knowledgeable on post-graduate rules and regulations do you find the Post-Graduate Administration?   1. Very knowledgeable 2. Moderately knowledgeable 3. Non-knowledgeable; specify why:…………….. 4. I don’t know | | | | |
| 33 | How do you find the competencies of staff within the Post-Graduate Administration?   1. Very competent 2. Moderately competent 3. Incompetent; specify why:…………….. 4. I don’t know | | | | |
| 34 | How do you find the friendliness of staff within the Post-Graduate Administration?   1. Very friendly 2. Moderately friendly 3. Unfriendly; specify why:…………….. 4. I don’t know | | | | |
| 35 | Has the Post-Graduate Administration negatively affected your post-graduate work in any way?   1. Yes; specify how:………………………… 2. No 3. I don’t know | | | | |
| 36 | Do you think anything could be done to improve the performance of the Post-Graduate Administration?   1. Yes; specify what could be done:………………………… 2. No 3. I don’t know | | | | |
| 37 | Were you exposed to any structured orientation programme (or briefing) for new post-graduate students when you started your post-graduate training at this university?   1. Yes 2. No 3. I don’t know/remember | | | | |
| 38 | Have any of your fellow-post-graduate students at this university have been exposed to any such programme?   1. Yes 2. No 3. I don’t know | | | | |
| 39 | Do you think that it would be useful with such a programme?   1. Yes; why?......... 2. No; why not:………. 3. I don’t know | | | | |
| 40 | Does this university have an association for post-graduate students?   1. Yes; why?......... 2. No; why not:………. 3. I don’t know | | | | |
| 41 | Do you think that it would be useful with such an association?   1. Yes; why?......... 2. No; why not:………. 3. I don’t know | | | | |
| 42 | Does this university have a bulletin for post-graduate students?   1. Yes [go to 44] 2. No 3. I don’t know | | | | |
| 43 | Do you think that it would be useful with such a bulletin?   1. Yes; why?................ 2. No; why not:…………… [go to 45] 3. I don’t know [go to 45] | | | | |
| 44 | Which topics would you like to see covered by a bulletin?   1. ……………. 2. ……………. 3. …………….…………………. | | | | |
| **E. UNIVERSITY FACILITIES AND SERVICES** | | | | | |
| 45 | Please express your opinion from your perspective as a post-graduate student about the facilities (items 1-18) at this university using the following scale from 1-4 (or 5 if you don’t know):  1. Strongly agree 2. Agree 3. Disagree 4. Strongly disagree 5. I don’t know  Answer ↓ | | | | |
|  | 1 | Internet accessibility for post-graduate students is satisfactory | | |  |
|  | 2 | Library facilities for post-graduate students is satisfactory | | |  |
|  | 3 | Opportunities to share libraries between departments/faculties is satisfactory | | |  |
|  | 4 | Opportunities to share libraries between institutions is satisfactory | | |  |
|  | 5 | Laboratories are adequately equipped for post-graduate training | | |  |
|  | 6 | Laboratories are adequately staffed for post-graduate training | | |  |
|  | 7 | Opportunities to share laboratories between departments/faculties is satisfactory | | |  |
|  | 8 | Opportunities to share laboratories between institutions is satisfactory | | |  |
|  | 9 | Availability of competent teachers for post-graduate training is satisfactory | | |  |
|  | 10 | Availability of relevant courses for post-graduate training is satisfactory | | |  |
|  | 11 | Advertisement of post-graduate courses offered by the university is satisfactory | | |  |
|  | 12 | Advertisement of post-graduate courses offered by other universities is satisfactory | | |  |
|  | 13 | Advertisement of post-graduate grant and scholarship opportunities is satisfactory | | |  |
|  | 14 | Management of post-graduate grants and scholarships is satisfactory | | |  |
|  | 15 | Opportunities for post-graduate students to pay exchange visits to other universities is satisfactory | | |  |
|  | 16 | Opportunities for post-graduate students to access courses at other universities is satisfactory | | |  |
|  | 17 | Support to career planning provided by the university/faculty/department is satisfactory | | |  |
|  | 18 | Career opportunities at the university is satisfactory | | |  |
| **F. MENTORSHIP FOR POST-GRADUATE STUDENTS** | | | | | |
| 46 | Do/did you have a written action plan describing what to do and when during the course of your post-graduate training?   1. Yes 2. No; why not?............ [go to 49] 3. I don’t know [go to 49] | | | | |
| 47 | Who developed this action plan?   1. Myself 2. My mentor(s) 3. Myself and my mentor(s) together 4. Others; specify whom:……………… 5. I don’t know | | | | |
| 48 | How well have you been able to adhere to this action plan?   1. Very well 2. Moderately well 3. Poorly; why?.............. 4. I don’t know | | | | |
| 49 | Are/were you assigned with one or more formal mentor(s) during your post-graduate training?   1. Yes; specify how many:…………. 2. No; specify why not:…………..…[go to 63] 3. I don’t know [go to 63] | | | | |
| 50 | How many of your formal mentors are/were affiliated to this university?   1. None [go to 54] 2. One 3. More than one; specify how many:……………. | | | | |
| 51 | Are/were any of these mentors permanent employees of the university?   1. Yes 2. No; describe your mentors’ relationship(s) with the university:……………….. 3. I don’t know | | | | |
| 52 | Are/were any of these mentors actively involved in conducting research?   1. Yes 2. No; specify why not, if known:………………… 3. I don’t know | | | | |
| 53 | What is/was the highest academic degree attained by the most senior of these mentors?   1. PhD/doctorate degree 2. Master degree 3. Other; specify:………………… 4. I don’t know | | | | |
| 54 | How many of your formal mentors come from another institution?   1. None 2. One; specify from where (country):………………….. 3. More than one; specify how many:……….and from where (country):……………………… | | | | |
| 55 | How well do you know the formal role of mentors in assisting post-graduate students?   1. Very well 2. Moderately well 3. Poorly [go to 57] | | | | |
| 56 | What are the sources of your knowledge about the formal role of mentors? | | | | |
|  | 1 | University or faculty handbook/guide | 1. Yes 2. No | | |
|  | 2 | Departmental handbook/guide | 1. Yes 2. No | | |
|  | 3 | University website | 1. Yes 2. No | | |
|  | 4 | Official briefing | 1. Yes 2. No | | |
|  | 5 | Informal discussions with university staff | 1. Yes 2. No | | |
|  | 6 | Informal discussions with fellow students | 1. Yes 2. No | | |
|  | 7 | Other; specify:……………………………. | 1. Yes 2. No | | |
| 57 | How do/did you find the friendliness of your internal mentor(s)?  [*Internal mentors* are mentors affiliated to this university]   1. Very friendly 2. Moderately friendly 3. Unfriendly; specify why:…………….. 4. I don’t know | | | | |
| 58 | How do/did you find your internal mentors’ level of commitment towards assisting you?   1. Very high 2. Moderately high 3. Low; specify why:…………….. 4. I don’t know | | | | |
| 59 | How do/did you find the usefulness of assistance received by your internal mentor(s)?   1. Very useful 2. Moderately useful 3. Useless; specify why:…………….. 4. I don’t know | | | | |
| 60 | How do/did you find the level of integrity (honesty and reliability) of your internal mentor(s)?   1. Very high 2. Moderately high 3. Low; specify why:………………. 4. I don’t know | | | | |
| 61 | How do/did you find the level of accessibility of your internal mentor(s)?   1. Easily accessible 2. Moderately accessible 3. Inaccessible; specify why:……………….. 4. I don’t know | | | | |
| 62 | Are/were you satisfied with the regularity of your contacts with your internal mentor(s)?   1. Yes 2. No; specify why not:…………..……. 3. I don’t know | | | | |
| 63 | Do you think anything could be done to improve the quality of support to post-graduate students at this university?   1. Yes; specify what could be done:………………………… 2. No 3. I don’t know | | | | |
| **G. TRAINING OF POST-GRADUATE STUDENTS** | | | | | |
| 64 | Are/were there any compulsory courses for you as a student enrolled in a post-graduate programme at this university?   1. Yes; specify how many courses are/were compulsory:………………… 2. No 3. I don’t know | | | | |
| 65 | Do you find it useful with compulsory courses for post-graduate students?   1. Yes; why?……………… 2. No; why not?......................... 3. I don’t know | | | | |
| 66 | Does this university offer a sufficiently comprehensive catalogue of post-graduate courses to suit your needs as a post-graduate student?   1. Yes [go to 68] 2. No 3. I don’t know [go to 68] | | | | |
| 67 | Which important courses do you find are missing?   1. ……………. 2. ……………. 3. …………….   …………………. | | | | |
| 68 | How many post-graduate courses have you taken at this university?……….. | | | | |
| 69 | Have you taken any post-graduate courses at this university which were of very high quality?   1. Yes; which ones?.................. 2. No [go to 71] 3. I don’t know [go to 71] | | | | |
| 70 | Why did you find this/these course(s) to be of very high quality?............. | | | | |
| 71 | Have you taken any post-graduate courses at this university which were of very poor quality?   1. Yes; which ones?.................. 2. No [go to 73] 3. I don’t know [go to 73] | | | | |
| 72 | Why did you find this/these course(s) of very poor quality?............. | | | | |
| 73 | Does this university offer a course in basic research methodology for post-graduate students?   1. Yes 2. No [go to 75] 3. I don’t know [go to 75] | | | | |
| 74 | Did you take this course?   1. Yes [go to 76] 2. No; why not?.......... | | | | |
| 75 | Where did you learn about basic research methodology?   1. Attending post-graduate course at another institution in this country; which one?......... 2. Attending post-graduate course abroad; where (country)?.......... 3. Was taught by mentor(s) 4. From undergraduate training 5. Other; specify where:……….. | | | | |
| 76 | Does this university offer a course in scientific writing skills for post-graduate students?   1. Yes 2. No [go to 78] 3. I don’t know [go to 78] | | | | |
| 77 | Did you take this course?   1. Yes 2. No; why not?.......... | | | | |
| 78 | How frequently have you been asked to evaluate the post-graduate courses that you have taken at this university?   1. Always 2. Very frequently 3. Sometimes 4. Rarely 5. Never | | | | |
| **H. IMPLEMENTATION AND DISSEMINATION OF RESEARCH** | | | | | |
| 79 | Are you (or will you be) conducting research as part of your post-graduate training?   1. Yes 2. No [go to 91] 3. I don’t know [go to 91] | | | | |
| 80 | Who defined the topic of your post-graduate research?   1. Myself 2. My mentor(s) 3. Myself and my mentor(s) together 4. Others; specify whom:……………… 5. I don’t know | | | | |
| 81 | How satisfied are you with the research topic?   1. Very satisfied 2. Moderately satisfied 3. Unsatisfied; specify why:……………… 4. I don’t know | | | | |
| 82 | How satisfied are you with the research process in general?   1. Very satisfied 2. Moderately satisfied 3. Unsatisfied; specify why:……………… 4. I don’t know | | | | |
| 83 | Have you discussed with your mentor(s) how to disseminate the scientific findings from your post-graduate project?   1. Yes; how?............. 2. No 3. I don’t know | | | | |
| 84 | At the current stage of your post-graduate work do you have any scientific findings ready for dissemination?   1. Yes 2. No; why not?.............[go to 90] 3. I don’t know [go to 90] | | | | |
| 85 | Have you published any of these findings in international scientific journals?   1. Yes; how many publications have you published?......... 2. No; why not?.......... [go to 87] 3. I don’t know [go to 87] | | | | |
| 86 | Are you the first author on this/all of these publications?   1. Yes 2. No; why not?:…………….. 3. I don’t know | | | | |
| 87 | Have you presented any of these findings at any departmental or faculty-level meetings or conferences?   1. Yes; where?…………. 2. No; why not?............. | | | | |
| 88 | Have you presented any of these findings at any national meetings or conferences?   1. Yes; where?……………… 2. No; why not?............. | | | | |
| 89 | Have you presented any of these findings at any international meetings or conferences?   1. Yes; where?………….. 2. No; why not?............. | | | | |
| 90 | Do you expect to publish the findings from your post-graduate project in international scientific journals?   1. Yes 2. No; why not?............ 3. I don’t know | | | | |
| 91 | If you have suggestions, which have not been addressed elsewhere in this questionnaire, for improving the post-graduate programme at this university, then please list them here: | | | | |
