# Supplementary material for: Needs assessment to strengthen capacity in water and sanitation research in Africa: experiences of the African SNOWS consortium
Source: Health Res Policy Syst. 2014 Dec 15;12:68. doi: 10.1186/1478-4505-12-68 (PMC4274706; doi:10.1186/1478-4505-12-68)
Supplement: Supplementary file 1 — Additional file 1: Zip file containing questionnaires used in the needs assessment. (ZIP 338 KB) [file 12961_2014_366_MOESM1_ESM.zip › SNOWS - Semi structured questionnaire - PhD students.docx]

SNOWS – Questionnaire for: *Current or recently graduated PhD fellows*

*Please, circle the correct code or fill in the response*

| **A. BACKGROUND AND PERSONAL INFORMATION** | | | | |
| --- | --- | --- | --- | --- |
| 1 | Date of filling the questionnaire (dd/mm/yyyy): | | | |
| 2 | At which of the following universities did you register for your PhD study?   1. Egerton University, Kenya 2. Kwame Nkrumah University of Science & Technology, Ghana 3. Mbara University of Science & Technology, Uganda 4. Tshwane University of Technology, South Africa 5. University of Gezira, Sudan 6. University of Venda, South Africa | | | |
| 3 | Category of respondent:   1. Currently registered as a PhD fellow; specify research topic:…………… 2. Recently graduated as a PhD (within the last two years); specify research topic:…………… | | | |
| 4 | Sex:   - - - 1. Male       2. Female | | | |
| 5 | Age (in years): | | | |
| 6 | Highest degree attained (below PhD level):   1. Master degree; subject:…………………......... 2. Bachelor degree; subject:……………………….. 3. Other; specify:………………………….. | | | |
| 7 | Awarding university (of highest degree attained below PhD-level); specify name and country:…………. | | | |
| 8 | Year of graduation (of highest degree attained below PhD-level): | | | |
| **B. PROJECT SPECIFIC DATA** | | | | |
| 9 | Department of registration of current or recently completed PhD: | | | |
| 10 | Date of official registration as PhD fellow (month and year): | | | |
| 11 | (Expected) date of submission of PhD thesis (month and year): | | | |
| 12 | (Expected) date of defence of PhD (month and year): | | | |
| 13 | Where does/did funding for your PhD project come from?   1. This university 2. A national funding agency 3. An international university 4. An international funding agency 5. Other; specify which one(s):…………………… 6. I don’t know | | | |
| 14 | Does/did the PhD budget make provisions for you to travel internationally for research meetings or conferences?   1. Yes 2. No 3. I don’t know | | | |
| 15 | Does/did the PhD budget make provisions for you to travel internationally for course participation?   1. Yes 2. No 3. I don’t know | | | |
| 16 | Did you travel internationally in connection with your PhD work?   1. Yes; specify where (country) and why (purpose):…………. 2. No | | | |
| 17 | Do/did you know the total amount of your PhD budget?   1. Yes; what is/was the approximate amount (remember currency)?................... 2. No | | | |
| **C. RULES AND REGULATIONS FOR PHD PROGRAMMES** | | | | |
| *The following questions refer to the university where you registered for your PhD study* | | | | |
| 18 | Has the university developed any written rules and regulations governing their PhD programmes?   1. Yes; specify what has been developed:………………………. 2. No [go to 20] 3. I don’t know [go to 20] | | | |
| 19 | Do you have a copy, hard or electronic, of any of these rules and regulations?   1. Yes; specify which documents you have:………………………… 2. No | | | |
| 20 | How well do you feel that you know the rules and regulations governing the PhD programmes at the university?   1. Very well 2. Well 3. Poorly 4. Very poorly [go to 22] | | | |
| 21 | What are the sources of your knowledge about the rules and regulations? | | | |
|  | 1 | University or faculty handbook/guide | 1. Yes 2. No | |
|  | 2 | Departmental handbook/guide | 1. Yes 2. No | |
|  | 3 | University website | 1. Yes 2. No | |
|  | 4 | Official briefing | 1. Yes 2. No | |
|  | 5 | Informal discussions with university staff | 1. Yes 2. No | |
|  | 6 | Informal discussions with other PhD fellows | 1. Yes 2. No | |
|  | 7 | Other; specify:……………………………. | 1. Yes 2. No | |
| 22 | How accessible are the rules and regulations?   1. Highly accessible [go to 25] 2. Moderately accessible [go to 24] 3. Difficult to access 4. I don’t know [go to 25] | | | |
| 23 | Please explain in which way access is difficult:………………….. | | | |
| 24 | Do you have any suggestions for making the rules and regulations more accessible to those who need them?   1. Yes; specify how:………………………… 2. No | | | |
|  |  |  |  |  |
|  |  |  |  |  |
|  |  |  |  |  |
| 25 | How do find the relevance of the rules and regulations to PhD fellows   1. Highly relevant 2. Moderately relevant 3. Irrelevant; specify why:……………… 4. I don’t know | | | |
| 26 | How do find the usefulness of the rules and regulations to PhD fellows   1. Highly useful 2. Moderately useful 3. Useless; specify why:……………… 4. I don’t know | | | |
| 27 | How do find the reasonableness of the rules and regulations to PhD fellows   1. Highly reasonable 2. Moderately reasonable 3. Unreasonable; specify why:…………….. 4. I don’t know | | | |
| 28 | What could be done to make the rules and regulations more relevant, useful and/or reasonable? | | | |
| 29 | To what extent have you personally used or referred to the rules and regulations?   1. Very frequently 2. Occasionally 3. Rarely; specify why:……………......... 4. Never; specify why:………………… | | | |
| **D. ADMINISTRATION AND ORGANISATION OF PHD PROGRAMMES** | | | | |
| *The following questions are addressing the administrative mechanisms for PhD programmes at this university. The responsible body within the university structure is referred to as the “PhD Administration”.* | | | | |
| 30 | How do you find the responsiveness of the PhD Administration towards meeting the needs of PhD fellows?   1. Highly responsive 2. Moderately responsive 3. Poorly responsive; specify why:…………….. 4. I don’t know | | | |
| 31 | How do you find the effectiveness of the PhD Administration in delivering services to PhD fellows?   1. Very effective 2. Moderately effective 3. Ineffective; specify why:…………….. 4. I don’t know | | | |
| 32 | How knowledgeable on PhD rules and regulations do you find the PhD Administration?   1. Very knowledgeable 2. Moderately knowledgeable 3. Non-knowledgeable; specify why:…………….. 4. I don’t know | | | |
| 33 | How do you find the competencies of staff within the PhD Administration?   1. Very competent 2. Moderately competent 3. Incompetent; specify why:…………….. 4. I don’t know | | | |
| 34 | How good is the PhD Administration at keeping records of relevance to PhD fellows?   1. Very good 2. Moderately good 3. Poor; specify why:…………….. 4. I don’t know | | | |
| 35 | How do you find the friendliness of staff within the PhD Administration?   1. Very friendly 2. Moderately friendly 3. Unfriendly; specify why:…………….. 4. I don’t know | | | |
| 36 | Has the PhD Administration negatively affected your PhD work in any way?   1. Yes; specify how:………………………… 2. No 3. I don’t know | | | |
| 37 | Do you think anything could be done to improve the performance of the PhD Administration?   1. Yes; specify what could be done:………………………… 2. No 3. I don’t know | | | |
| 38 | Were you exposed to any structured orientation programme (or briefing) for new PhD fellows when you started your PhD study at this university?   1. Yes 2. No [go to 40] 3. I don’t know/remember[go to 40] | | | |
| 39 | How satisfied were you with this orientation programme?   1. Very satisfied 2. Moderately satisfied 3. Un-satisfied; why?................ 4. I don’t know/remember | | | |
| 41 | Have any of your associated PhD fellows at this university have been exposed to any such programme?   1. Yes 2. No 3. I don’t know | | | |
| 42 | Do you think that it would be useful with such a programme?   1. Yes; why?......... 2. No; why not:………. 3. I don’t know | | | |
| 43 | Does this university have an association for PhD fellows?   1. Yes 2. No [go to 45] 3. I don’t know [go to 45] | | | |
| 44 | How satisfied are/were you with the function of this association?   1. Very satisfied 2. Moderately satisfied 3. Un-satisfied; why?................ 4. I don’t know | | | |
| 45 | Do you think that it would be useful with such an association?   1. Yes; why?......... 2. No; why not:………. 3. I don’t know | | | |
| 46 | Does this university have a bulletin for PhD fellows?   1. Yes 2. No [go to 48] 3. I don’t know [go to 48] | | | |
| 47 | How satisfied are/were you with the quality of this bulletin?   1. Very satisfied [go to 50] 2. Moderately satisfied [go to 50] 3. Un-satisfied; why?................ [go to 50] 4. I don’t know [go to 50] | | | |
| 48 | Do you think that it would be useful with such a bulletin?   1. Yes; why?................ 2. No; why not:…………… 3. I don’t know | | | |
| 49 | Which topics would you like to see covered by a bulletin?   1. ……………. 2. ……………. 3. …………….   …………………. | | | |
| **E. UNIVERSITY FACILITIES AND SERVICES** | | | | |
| 50 | Please express your opinion from your perspective as a PhD fellow about the facilities (items 1-18) at this university using the following scale from 1-4 (or 5 if you don’t know):  1. Strongly agree 2. Agree 3. Disagree 4. Strongly disagree 5. I don’t know  Answer ↓ | | | |
|  | 1 | Internet accessibility for PhD fellows is satisfactory | |  |
|  | 2 | Library facilities for PhD fellows is satisfactory | |  |
|  | 3 | Opportunities to share libraries between departments/faculties is satisfactory | |  |
|  | 4 | Opportunities to share libraries between institutions is satisfactory | |  |
|  | 5 | Laboratories are adequately equipped for PhD research | |  |
|  | 6 | Laboratories are adequately staffed for PhD research | |  |
|  | 7 | Opportunities to share laboratories between departments/faculties is satisfactory | |  |
|  | 8 | Opportunities to share laboratories between institutions is satisfactory | |  |
|  | 9 | Availability of competent teachers for PhD-level training is satisfactory | |  |
|  | 10 | Availability of relevant courses for PhD-level training is satisfactory | |  |
|  | 11 | Advertisement of PhD-level courses offered by the university is satisfactory | |  |
|  | 12 | Advertisement of PhD-level courses offered by other universities is satisfactory | |  |
|  | 13 | Advertisement of PhD grant and scholarship opportunities is satisfactory | |  |
|  | 14 | Management of PhD grants and scholarships is satisfactory | |  |
|  | 15 | Opportunities for PhD fellows to pay exchange visits to other universities is satisfactory | |  |
|  | 16 | Opportunities for PhD fellows to access courses at other universities is satisfactory | |  |
|  | 17 | Support to career planning provided by the university/faculty/department is satisfactory | |  |
|  | 18 | Career opportunities at the university is satisfactory | |  |
| **F. SUPERVISION OF PHD FELLOWS** | | | | |
| 51 | Who defined the topic of your PhD project? | | | |
|  | 1 | Myself | 1. Yes 2. No | |
|  | 2 | My supervisor(s) | 1. Yes 2. No | |
|  | 3 | The department | 1. Yes 2. No | |
|  | 4 | Others; specify whom:……………… | 1. Yes 2. No | |
|  | 5 | I don’t know | 1. Yes 2. No | |
| 52 | Who defined the research protocol of your PhD project? | | | |
|  | 1 | Myself | 1. Yes 2. No | |
|  | 2 | My supervisor(s) | 1. Yes 2. No | |
|  | 3 | The department | 1. Yes 2. No | |
|  | 4 | Others; specify whom:……………… | 1. Yes 2. No | |
|  | 5 | I don’t know | 1. Yes 2. No | |
| 53 | Do/did you have a written action plan describing what to do and when during the course of your PhD project?   1. Yes 2. No; why not?............ [go to 56] 3. I don’t know [go to 56] | | | |
| 54 | Who developed this action plan? | | | |
|  | 1 | Myself | 1. Yes 2. No | |
|  | 2 | My supervisor(s) | 1. Yes 2. No | |
|  | 3 | The department | 1. Yes 2. No | |
|  | 4 | Others; specify whom:……………… | 1. Yes 2. No | |
|  | 5 | I don’t know | 1. Yes 2. No | |
| 55 | How well have you been able to adhere to this action plan?   1. Very well 2. Moderately well 3. Poorly; why?.............. 4. I don’t know | | | |
| 56 | Are/were you assigned with one or more formal supervisor(s) during your PhD work?   1. Yes; specify how many:…………. 2. No; specify why not:…………..…[go to 62] 3. I don’t know [go to 62] | | | |
| 57 | How many of your formal supervisors are/were affiliated to this university?   1. None [go to 61] 2. One 3. More than one; specify how many:……………. | | | |
| 58 | Are/were any of these supervisors permanent employees of the university?   1. Yes 2. No; describe your supervisors’ relationship(s) with the university:……………….. 3. I don’t know | | | |
| 59 | Are/were any of these supervisors actively involved in conducting research?   1. Yes 2. No; specify why not, if known:………………… 3. I don’t know | | | |
| 60 | What is/was the highest academic degree attained by the most senior of these supervisors?   1. PhD/doctorate degree 2. Master degree 3. Other; specify:………………… 4. I don’t know | | | |
| 61 | How many of your formal supervisors come from another institution?   1. None 2. One; specify from where (country):………………….. 3. More than one; specify how many:……….and from where (country):……………………… | | | |
| 62 | How well do you know the formal role of PhD supervisor(s) in providing supervision?   1. Very well 2. Moderately well 3. Poorly [go to 64] | | | |
| 63 | What are the sources of your knowledge about the formal role of supervisors? | | | |
|  | 1 | University or faculty handbook/guide | 1. Yes 2. No | |
|  | 2 | Departmental handbook/guide | 1. Yes 2. No | |
|  | 3 | University website | 1. Yes 2. No | |
|  | 4 | Official briefing | 1. Yes 2. No | |
|  | 5 | Informal discussions with university staff | 1. Yes 2. No | |
|  | 6 | Informal discussions with fellow fellows | 1. Yes 2. No | |
|  | 7 | Other; specify:……………………………. | 1. Yes 2. No | |
| 64 | How do/did you find the friendliness of your internal supervisor(s)?  [*Internal supervisors* are supervisors affiliated to this university]   1. Very friendly 2. Moderately friendly 3. Unfriendly; specify why:…………….. 4. I don’t know | | | |
| 65 | How do/did you find your internal supervisors’ level of commitment towards providing you with supervision?   1. Very high 2. Moderately high 3. Low; specify why:…………….. 4. I don’t know | | | |
| 66 | How do/did you find the usefulness of supervision received by your internal supervisor(s)?   1. Very useful 2. Moderately useful 3. Useless; specify why:…………….. 4. I don’t know | | | |
| 67 | How do/did you find the level of integrity (honesty and reliability) of your internal supervisor(s)?   1. Very high 2. Moderately high 3. Low; specify why:………………. 4. I don’t know | | | |
| 68 | How do/did you find the level of accessibility of your internal supervisor(s)?   1. Easily accessible 2. Moderately accessible 3. Inaccessible; specify why:……………….. 4. I don’t know | | | |
| 69 | How do/did you find the scientific competencies of your internal supervisor(s)?   1. Very high 2. Moderately high 3. Low 4. I don’t know | | | |
| 70 | How satisfied are/were you with the regularity of your contacts with your internal supervisor(s)?   1. Very satisfied 2. Moderately satisfied 3. Un-satisfied; specify why?…………..……. 4. I don’t know | | | |
| 71 | Do you think anything could be done to improve the quality of supervision at this university?   1. Yes; specify what could be done:………………………… 2. No 3. I don’t know | | | |
| **G. TRAINING OF PHD FELLOWS** | | | | |
| 72 | Are/were there any compulsory courses for you as a PhD fellow enrolled in a PhD programme at this university?   1. Yes; specify which courses are/were compulsory:………………… 2. No 3. I don’t know | | | |
| 73 | Do you find it useful with compulsory courses for PhD fellows?   1. Yes; why?……………… 2. No; why not?......................... 3. I don’t know | | | |
| 74 | Does this university offer a sufficiently comprehensive catalogue of PhD-level courses to suit your needs as a PhD fellow?   1. Yes [go to 76] 2. No 3. I don’t know [go to 76] | | | |
| 75 | Which important courses do you find are missing?   1. ……………. 2. ……………. 3. …………….   …………………. | | | |
| 76 | How many PhD-level courses have you taken at this university?:……….. [if none, go to 82] | | | |
| 77 | What are the (approximate) titles or topics of these courses?   1. ……………. 2. ……………. 3. …………….   …………………. | | | |
| 78 | Have you taken any PhD-level courses at this university which were of very high quality?   1. Yes; which ones?.................. 2. No [go to 80] 3. I don’t know [go to 80] | | | |
| 79 | Why did you find this/these course(s) to be of very high quality?............. | | | |
| 80 | Have you taken any PhD-level courses at this university which were of very poor quality?   1. Yes; which ones?.................. 2. No [go to 82] 3. I don’t know [go to 82] | | | |
| 81 | Why did you find this/these course(s) of very poor quality?............. | | | |
| 82 | Does this university offer a course in basic research methodology for PhD fellows?   1. Yes 2. No [go to 84] 3. I don’t know [go to 84] | | | |
| 83 | Did you take this course?   1. Yes [go to 85] 2. No; why not?.......... | | | |
| 84 | Where did you learn about basic research methodology? | | | |
|  | 1 | Attending PhD course at another institution in this country; which one?......... | 1. Yes 2. No | |
|  | 2 | Attending PhD course abroad; where (country)?.......... | 1. Yes 2. No | |
|  | 3 | Was taught informally by supervisor(s) | 1. Yes 2. No | |
|  | 4 | From undergraduate training | 1. Yes 2. No | |
|  | 5 | From post-graduate training | 1. Yes 2. No | |
|  | 6 | Other; specify where:……….. | 1. Yes 2. No | |
| 85 | Does this university offer a course in scientific writing skills for PhD fellows?   1. Yes 2. No [go to 87] 3. I don’t know [go to 87] | | | |
| 86 | Did you take this course?   1. Yes 2. No; why not?.......... | | | |
| 87 | How frequently have you been asked to evaluate the PhD-level courses that you have taken at this university?   1. Always 2. Very frequently 3. Sometimes 4. Rarely 5. Never | | | |
| **H. DISSEMINATION OF RESEARCH FINDINGS** | | | | |
| 88 | Have you discussed with your supervisor(s) how to disseminate the scientific findings from your PhD project?   1. Yes; how?............. 2. No 3. I don’t know | | | |
| 89 | At the current stage of your PhD work do you have any scientific findings ready for dissemination?   1. Yes 2. No; why not?.............[go to 95] 3. I don’t know [go to 95] | | | |
| 90 | Have you published any of these findings in international scientific journals?   1. Yes; how many publications have you published?......... 2. No; why not?.......... [go to 92] 3. I don’t know [go to 92] | | | |
| 91 | Are you the first author on this/all of these publications?   1. Yes 2. No; why not?:…………….. 3. I don’t know | | | |
| 92 | Have you presented any of these findings at any departmental or faculty-level meetings or conferences?   1. Yes; where?…………. 2. No; why not?............. | | | |
| 93 | Have you presented any of these findings at any national meetings or conferences?   1. Yes; where?……………… 2. No; why not?............. | | | |
| 94 | Have you presented any of these findings at any international meetings or conferences?   1. Yes; where?………….. 2. No; why not?............. | | | |
| 95 | Do you expect to publish the findings from your PhD project in international scientific journals?   1. Yes 2. No; why not?............ 3. I don’t know | | | |
| 96 | Does publishing of scientific papers contribute to the final evaluation of a PhD study at this university?   1. Yes 2. No 3. I don’t know | | | |
| 97 | Do you think that publishing of scientific papers should contribute to the final evaluation of a PhD at this university?   1. Yes; why?..................... 2. No; why?.............. 3. I don’t know | | | |
| 98 | If you have suggestions, which have not been addressed elsewhere in this questionnaire, for improving the PhD programme at this university, then please list them here: | | | |
