# Supplementary material for: Needs assessment to strengthen capacity in water and sanitation research in Africa: experiences of the African SNOWS consortium
Source: Health Res Policy Syst. 2014 Dec 15;12:68. doi: 10.1186/1478-4505-12-68 (PMC4274706; doi:10.1186/1478-4505-12-68)
Supplement: Supplementary file 1 — Additional file 1: Zip file containing questionnaires used in the needs assessment. (ZIP 338 KB) [file 12961_2014_366_MOESM1_ESM.zip › SNOWS - Semi structured questionnaire - Academics.docx]

SNOWS – Questionnaire for: *Academic personnel*

*Please, circle the correct code or fill in the response*

| **A. BACKGROUND AND PERSONAL INFORMATION** | | | | | | | |
| --- | --- | --- | --- | --- | --- | --- | --- |
| 1 | Date of filling the questionnaire (dd/mm/yyyy): | | | | | | |
| 2 | At which of the following universities do you work?   1. Egerton University, Kenya 2. Kwame Nkrumah University of Science & Technology, Ghana 3. Mbara University of Science & Technology, Uganda 4. Tshwane University of Technology, South Africa 5. University of Gezira, Sudan 6. University of Venda, South Africa | | | | | | |
| 3 | When did you start working at this university (month and year)? | | | | | | |
| 4 | Name of department where you work:…………………………………….. | | | | | | |
| 5 | When did you start working at this department (month and year)? | | | | | | |
| 6 | What is your academic position at the university?   1. Professor 2. Associate professor 3. Senior lecturer 4. Lecturer 5. Assistant lecturer 6. Other; specify:……………….. | | | | | | |
| 7 | What are your duties and responsibilities at the university? | | | | | | |
|  | 1 | | Supervising PhD fellows | | | 1. Yes 2. No | |
|  | 2 | | Mentoring Master-level students | | | 1. Yes 2. No | |
|  | 3 | | Teaching/lecturing | | | 1. Yes 2. No | |
|  | 4 | | Conducting research | | | 1. Yes 2. No | |
|  | 5 | | Doing administrative work for the department | | | 1. Yes 2. No | |
|  | 6 | | Other; specify:……………………………. | | | 1. Yes 2. No | |
| 8 | Sex:   - - - 1. Male       2. Female | | | | | | |
| 9 | Age (in years): | | | | | | |
| 10 | Highest degree attained:   1. PhD degree; subject:……………… 2. Master degree; subject:…………………......... 3. Bachelor degree; subject:……………………….. 4. Other; specify:………………………….. | | | | | | |
| 11 | Awarding university (of highest degree attained); specify name and country:…………. | | | | | | |
| 12 | Year of graduation (of highest degree attained): | | | | | | |
| **B. PHD PROGRAMMES** | | | | | | | |
| *The following questions relate to the PhD programme(s) at the university, faculty and department where you work* | | | | | | | |
| 13 | Does this faculty have one or more PhD programmes?   1. Yes 2. No [go to 68] 3. I don’t know [go to 68] | | | | | | |
| 14 | Does this department have any PhD fellows registered in any such programme?   1. Yes 2. No; why not?.............. 3. I don’t know | | | | | | |
| 15 | Are you actively involved in any such PhD programme?   1. Yes; in what way?.............. 2. No; why not?............... | | | | | | |
| 16 | How well do you feel that you know the rules and regulations governing the PhD programmes at this faculty?   1. Very well 2. Well 3. Poorly 4. Very poorly [go to 19] | | | | | | |
| 17 | What are the sources of your knowledge about the PhD-related rules and regulations? | | | | | | |
|  | 1 | | | | University or faculty handbook/guide | 1. Yes 2. No | |
|  | 2 | | | | Departmental handbook/guide | 1. Yes 2. No | |
|  | 3 | | | | University website | 1. Yes 2. No | |
|  | 4 | | | | Official briefing | 1. Yes 2. No | |
|  | 5 | | | | Informal discussions with university staff | 1. Yes 2. No | |
|  | 6 | | | | Informal discussions with PhD fellows | 1. Yes 2. No | |
|  | 7 | | | | Other; specify:……………………………. | 1. Yes 2. No | |
| 18 | To what extent have you personally used or referred to the PhD-related rules and regulations?   1. Very frequently 2. Occasionally 3. Rarely; specify why:……………......... 4. Never; specify why:………………… | | | | | | |
| *The following questions relate to communication* | | | | | | | |
| 19 | How satisfied are you with the communication about PhD related issues between administrative bodies and academic staff at the university?   1. Very satisfied 2. Moderately satisfied 3. Un-satisfied; why?…………... 4. I don’t know | | | | | | |
| 20 | How satisfied are you with the communication about PhD related issues within the group of academic staff members at the university?   1. Very satisfied 2. Moderately satisfied 3. Un-satisfied; why?…………... 4. I don’t know | | | | | | |
| 21 | Does this faculty or department have a structured orientation programme (or briefing) for new PhD fellows?   1. Yes 2. No [go to 24] 3. I don’t know [go to 24] | | | | | | |
| 22 | Have you been involved in providing new PhD fellows with such an orientation or briefing?   1. Yes 2. No; why not?......................... | | | | | | |
| 23 | How satisfied are you with this orientation programme?   1. Very satisfied [go to 25] 2. Moderately satisfied [go to 25] 3. Un-satisfied; specify why?................ [go to 25] 4. I don’t know [go to 25] | | | | | | |
| 24 | Do you think that it would be useful with such an orientation programme?   1. Yes; why?......... 2. No; why not?………. 3. I don’t know | | | | | | |
| 25 | Does this university or faculty have an association for PhD fellows?   1. Yes 2. No [go to 27] 3. I don’t know [go to 27] | | | | | | |
| 26 | How satisfied are/were you with the function of this association?   1. Very satisfied [go to 28] 2. Moderately satisfied [go to 28] 3. Un-satisfied; why?................ [go to 28] 4. I don’t know[go to 28] | | | | | | |
| 27 | Do you think that it would be useful with such an association?   1. Yes; why?......... 2. No; why not?………. 3. I don’t know | | | | | | |
| 28 | Does this university or faculty have a bulletin for PhD fellows?   1. Yes 2. No [go to 30] 3. I don’t know [go to 30] | | | | | | |
| 29 | How satisfied are you with the quality of this bulletin?   1. Very satisfied [go to 31] 2. Moderately satisfied [go to 31] 3. Un-satisfied; why?................ [go to 31] 4. I don’t know [go to 31] | | | | | | |
| 30 | Do you think that it would be useful with such a bulletin?   1. Yes; why?................ 2. No; why not?…………… 3. I don’t know | | | | | | |
| *The following questions relate to facilities and services available for PhD fellows* | | | | | | | |
| 31 | Please express your opinion from your position as a supervisor and/or lecturer for PhD fellows about the facilities (items 1-18) at this university using the following scale from 1-4 (or 5 if you don’t know):  1. Strongly agree 2. Agree 3. Disagree 4. Strongly disagree 5. I don’t know  Answer ↓ | | | | | | |
|  | 1 | | | | Internet accessibility for PhD fellows is satisfactory | |  |
|  | 2 | | | | Library facilities for PhD fellows is satisfactory | |  |
|  | 3 | | | | Opportunities to share libraries between departments/faculties is satisfactory | |  |
|  | 4 | | | | Opportunities to share libraries between institutions is satisfactory | |  |
|  | 5 | | | | Laboratories are adequately equipped for PhD research | |  |
|  | 6 | | | | Laboratories are adequately staffed for PhD research | |  |
|  | 7 | | | | Opportunities to share laboratories between departments/faculties is satisfactory | |  |
|  | 8 | | | | Opportunities to share laboratories between institutions is satisfactory | |  |
|  | 9 | | | | Availability of competent teachers for PhD-level training is satisfactory | |  |
|  | 10 | | | | Availability of relevant courses for PhD-level training is satisfactory | |  |
|  | 11 | | | | Advertisement of PhD-level courses offered by the university is satisfactory | |  |
|  | 12 | | | | Advertisement of PhD-level courses offered by other universities is satisfactory | |  |
|  | 13 | | | | Advertisement of PhD grant and scholarship opportunities is satisfactory | |  |
|  | 14 | | | | Management of PhD grants and scholarships is satisfactory | |  |
|  | 15 | | | | Opportunities for PhD fellows to pay exchange visits to other universities is satisfactory | |  |
|  | 16 | | | | Opportunities for PhD fellows to access courses at other universities is satisfactory | |  |
|  | 17 | | | | Support provided by the university to individual career planning for PhD fellows is satisfactory | |  |
|  | 18 | | | | Career opportunities for PhD graduates at the university are satisfactory | |  |
| *The following questions relate to the role and function of PhD supervisors* | | | | | | | |
| 32 | Have you been a formal supervisor or co-supervisor of any PhD fellows at this university?   1. Yes; how many?................ 2. No; why not?................[go to 49] | | | | | | |
| 33 | Who defined the research topics in the PhD projects that you have supervised? | | | | | | |
|  | 1 | | | The PhD fellow | | 1. Yes 2. No | |
|  | 2 | | | The supervisor(s) | | 1. Yes 2. No | |
|  | 3 | | | The department | | 1. Yes 2. No | |
|  | 4 | | | Others; specify whom:……………… | | 1. Yes 2. No | |
| 34 | Who developed the research protocols in the PhD projects that you have supervised? | | | | | | |
|  | 1 | | | The PhD fellow | | 1. Yes 2. No | |
|  | 2 | | | The supervisor(s) | | 1. Yes 2. No | |
|  | 3 | | | The department | | 1. Yes 2. No | |
|  | 4 | | | Others; specify whom:……………… | | 1. Yes 2. No | |
| 35 | Do PhD fellows have a written action plan describing what to do and when during the course of a PhD project?   1. Yes 2. No; why not?............ [go to 37] 3. I don’t know [go to 37] | | | | | | |
| 36 | Who developed this action plan in the PhD projects that you have supervised? | | | | | | |
|  | 1 | | | The PhD fellow | | 1. Yes 2. No | |
|  | 2 | | | The supervisor(s) | | 1. Yes 2. No | |
|  | 3 | | | The department | | 1. Yes 2. No | |
|  | 4 | | | Others; specify whom:……………… | | 1. Yes 2. No | |
| 37 | How well do you know the formal role of PhD supervisor(s) in providing supervision?   1. Very well 2. Moderately well 3. Poorly [go to 39] | | | | | | |
| 38 | What are the sources of your knowledge about the formal role of supervisors? | | | | | | |
|  | 1 | | | | University or faculty handbook/guide | 1. Yes 2. No | |
|  | 2 | | | | Departmental handbook/guide | 1. Yes 2. No | |
|  | 3 | | | | University website | 1. Yes 2. No | |
|  | 4 | | | | Official briefing | 1. Yes 2. No | |
|  | 5 | | | | Informal discussions with university staff | 1. Yes 2. No | |
|  | 6 | | | | Informal discussions with PhD fellows | 1. Yes 2. No | |
|  | 7 | | | | Other; specify:……………………………. | 1. Yes 2. No | |
| 39 | How satisfied are you with your own competencies in providing supervision to PhD fellows?   1. Very satisfied 2. Moderately satisfied 3. Un-satisfied | | | | | | |
| 40 | Have you received any formal training in how to supervise PhD fellows?   1. Yes; where did you receive this training (name of institution and country)?...................[go to 42] 2. No | | | | | | |
| 41 | Would you be interested in receiving formal training in how to supervise PhD fellows?   1. Yes 2. No; why not?................... | | | | | | |
| 42 | Are there any standards or rules for how much time you should spend on supervising your PhD fellows?   1. Yes; specify the amount of time in hours per week:………….. 2. No 3. I don’t know | | | | | | |
| 43 | How do you manage the time that you spend on supervising your PhD fellows? | | | | | | |
|  | 1 | | | I keep specific “office opening hours” for PhD supervision | | 1. Yes 2. No | |
|  | 2 | | | I offer supervision based on appointments requested by PhD fellows | | 1. Yes 2. No | |
|  | 3 | | | I don’t; my office is always open for PhD fellows when I am around | | 1. Yes 2. No | |
|  | 4 | | | Others; specify how:……………… | | 1. Yes 2. No | |
| 44 | How satisfied are you with the regularity of your contacts with your PhD fellows?   1. Very satisfied 2. Moderately satisfied 3. Un-satisfied; specify why?......................... 4. I don’t know | | | | | | |
| *The following questions relate to dissemination of PhD research findings* | | | | | | | |
| 45 | Do you normally discuss with your PhD fellows how to disseminate the scientific findings from their PhD projects?   1. Yes 2. No; why not?..................... | | | | | | |
| 46 | How many of your own PhD fellows have published at least some of their research findings in international peer reviewed journals (either before or after defending their PhD thesis)?   1. None 2. Between 1-25% 3. Between 25-50% 4. Between 50-75% 5. Between 75-99% 6. All of them 7. I don’t know | | | | | | |
| 47 | Does publishing of scientific papers contribute to the final evaluation of a PhD study at this faculty?   1. Yes 2. No 3. I don’t know | | | | | | |
| 48 | Do you think that publishing of scientific papers should contribute to the final evaluation of a PhD at this university?   1. Yes; why?..................... 2. No; why?.............. 3. I don’t know | | | | | | |
| *The following questions relate to PhD courses and teaching* | | | | | | | |
| 49 | Have you been involved in teaching PhD fellows at this faculty during the last couple of years?   1. Yes; specify in how many courses you taught:………………… 2. No; why not?.....................[go to 53] | | | | | | |
| 50 | How satisfied are/were you with teaching PhD fellows?   1. Very satisfied 2. Moderately satisfied 3. Un-satisfied; specify why?................... | | | | | | |
| 51 | How satisfied are you with the level of recognition provided by the university for teaching PhD fellows?   1. Very satisfied 2. Moderately satisfied 3. Un-satisfied; specify why?................... | | | | | | |
| 52 | How satisfied are you with your own teaching skills?   1. Very satisfied 2. Moderately satisfied 3. Un-satisfied | | | | | | |
| 53 | Have you received any formal training in how to teach?   1. Yes; where (name of training institution and country)…………..[go to 55] 2. No | | | | | | |
| 54 | Would you be interested in receiving formal training in how to teach?   1. Yes 2. No; why not?................... | | | | | | |
| 55 | Are there any compulsory courses for PhD fellows enrolled in a PhD programme at this faculty or department?   1. Yes; specify how many courses are compulsory:………………… 2. No 3. I don’t know | | | | | | |
| 56 | Does this faculty or department offer a sufficiently comprehensive catalogue of PhD courses?   1. Yes [go to 58] 2. No 3. I don’t know [go to 58] | | | | | | |
| 57 | Which important courses do you find are missing?   1. ……………. 2. ……………. 3. ……………………………………………………… | | | | | | |
| 58 | Does the university, faculty or department encourage academic staff to develop new courses for PhD fellows?   1. Yes 2. No 3. I don’t know | | | | | | |
| 59 | Have you been involved in developing any new courses for PhD fellows within the last couple of years?   1. Yes 2. No; why not?....................... | | | | | | |
| 60 | Are there mechanisms in place at the university for reviewing the relevance and quality of courses offered to PhD fellows?   1. Yes 2. No [go to 65] 3. I don’t know [go to 65] | | | | | | |
| 61 | How satisfied are you with the effectiveness of these reviews?   1. Very satisfied 2. Moderately satisfied 3. Un-satisfied; specify why?................... | | | | | | |
| 62 | How are the reviews carried out?   1. As internal reviews by the department(s) 2. As internal reviews by the faculty 3. As external reviews by consultancy firms or other training institutions 4. Other; specify how?............. 5. I don’t know | | | | | | |
| 63 | How often are the reviews carried out?   1. About once every year 2. About once every two years 3. Other; specify how many years between each review?............. 4. I don’t know | | | | | | |
| 64 | Are PhD fellows involved in the evaluation of courses?   1. Yes 2. No 3. I don’t know | | | | | | |
| 65 | Does this faculty or department offer a course in basic research methodology for PhD fellows?   1. Yes 2. No [go to 67] 3. I don’t know | | | | | | |
| 66 | Where do PhD fellows normally learn about basic research methodology? | | | | | | |
|  | 1 | | | Attending PhD course at another institution in this country; which one?......... | | 1. Yes 2. No | |
|  | 2 | | | Attending PhD course abroad; where (country)?.......... | | 1. Yes 2. No | |
|  | 3 | | | Taught informally by supervisor(s) | | 1. Yes 2. No | |
|  | 4 | | | From undergraduate training | | 1. Yes 2. No | |
|  | 5 | | | From post-graduate training | | 1. Yes 2. No | |
|  | 6 | | | Other; specify where:……….. | | 1. Yes 2. No | |
|  | 7 | | | I don’t know | | 1. Yes 2. No | |
| 67 | If you have suggestions, which have not been addressed elsewhere in this questionnaire, for improving the PhD programme at this university, then please list them here: | | | | | | |
| **C. POST-GRADUATE PROGRAMMES** | | | | | | | |
| The following questions relate to the post-graduate programme(s) at the university, faculty and department where you work | | | | | | | |
| 68 | Does this faculty have one or more post-graduate programmes?   1. Yes 2. No [go to 116] 3. I don’t know [go to 116] | | | | | | |
| 69 | Does this department have any students enrolled in any such post-graduate programme?   1. Yes 2. No; why not?............... 3. I don’t know | | | | | | |
| 70 | Are you actively involved in any such post-graduate programmes?   1. Yes; in what way?.............. 2. No; why not?............... | | | | | | |
| 71 | How well do you know the rules and regulations governing the post-graduate programmes at this university?   1. Very well 2. Well 3. Poorly 4. Very poorly [go to 74] | | | | | | |
| 72 | What are the sources of your knowledge about the rules and regulations for post-graduate students? | | | | | | |
|  | 1 | | | University or faculty handbook/guide | | 1. Yes 2. No | |
|  | 2 | | | Departmental handbook/guide | | 1. Yes 2. No | |
|  | 3 | | | University website | | 1. Yes 2. No | |
|  | 4 | | | Official briefing | | 1. Yes 2. No | |
|  | 5 | | | Informal discussions with university staff | | 1. Yes 2. No | |
|  | 6 | | | Informal discussions with post-graduate students | | 1. Yes 2. No | |
|  | 7 | | | Other; specify:……………………………. | | 1. Yes 2. No | |
| 73 | To what extent have you personally used or referred to the rules and regulations for post-graduate students?   1. Very frequently 2. Occasionally 3. Rarely; specify why:……………......... 4. Never; specify why:………………… | | | | | | |
| *The following questions relate to communication* | | | | | | | |
| 74 | How satisfied are you with the communication about issues related to post-graduate programmes between administrative bodies and academic staff at the university?   1. Very satisfied 2. Moderately satisfied 3. Un-satisfied; why?…………... 4. I don’t know | | | | | | |
| 75 | How satisfied are you with the communication about issues related to post-graduate programmes within the group of academic staff members at the university?   1. Very satisfied 2. Moderately satisfied 3. Un-satisfied; why?…………... 4. I don’t know | | | | | | |
| 76 | Does this faculty or department have a structured orientation programme (or briefing) for new post-graduate students?   1. Yes 2. No [go to 79] 3. I don’t know [go to 79] | | | | | | |
| 77 | Have you been involved in providing new post-graduate students with such an orientation or briefing?   1. Yes 2. No; why not?......................... | | | | | | |
| 78 | How satisfied are you with this orientation programme?   1. Very satisfied [go to 80] 2. Moderately satisfied [go to 80] 3. Un-satisfied; specify why?................ [go to 80] 4. I don’t know [go to 80] | | | | | | |
| 79 | Do you think that it would be useful with such an orientation programme?   1. Yes; why?......... 2. No; why not?………. 3. I don’t know | | | | | | |
| 80 | Does this university have an association for post-graduate students?   1. Yes 2. No [go to 82] 3. I don’t know [go to 82] | | | | | | |
| 81 | How satisfied are/were you with the function of this association?   1. Very satisfied [go to 83] 2. Moderately satisfied [go to 83] 3. Un-satisfied; why?................ [go to 83] 4. I don’t know[go to 83] | | | | | | |
| 82 | Do you think that it would be useful with such an association?   1. Yes; why?......... 2. No; why not?………. 3. I don’t know | | | | | | |
| 83 | Does this university have a bulletin for post-graduate students?   1. Yes 2. No [go to 85] 3. I don’t know [go to 85] | | | | | | |
| 84 | How satisfied are you with the quality of this bulletin?   1. Very satisfied [go to 86] 2. Moderately satisfied [go to 86] 3. Un-satisfied; why?................ [go to 86] 4. I don’t know [go to 86] | | | | | | |
| 85 | Do you think that it would be useful with such a bulletin?   1. Yes; why?................ 2. No; why not?…………… 3. I don’t know | | | | | | |
| *The following questions relate to facilities and services available for post-graduate students* | | | | | | | |
| 86 | Please express your opinion from your position as a mentor and/or lecturer for post-graduate students about the facilities (items 1-18) at this university using the following scale from 1-4 (or 5 if you don’t know):  1. Strongly agree 2. Agree 3. Disagree 4. Strongly disagree 5. I don’t know  Answer ↓ | | | | | | |
|  | 1 | | | Internet accessibility for post-graduate students is satisfactory | | |  |
|  | 2 | | | Library facilities for post-graduate students is satisfactory | | |  |
|  | 3 | | | Opportunities to share libraries between departments/faculties is satisfactory | | |  |
|  | 4 | | | Opportunities to share libraries between institutions is satisfactory | | |  |
|  | 5 | | | Laboratories are adequately equipped for post-graduate training | | |  |
|  | 6 | | | Laboratories are adequately staffed for post-graduate training | | |  |
|  | 7 | | | Opportunities to share laboratories between departments/faculties is satisfactory | | |  |
|  | 8 | | | Opportunities to share laboratories between institutions is satisfactory | | |  |
|  | 9 | | | Availability of competent teachers for post-graduate training is satisfactory | | |  |
|  | 10 | | | Availability of relevant courses for post-graduate training is satisfactory | | |  |
|  | 11 | | | Advertisement of post-graduate courses offered by the university is satisfactory | | |  |
|  | 12 | | | Advertisement of post-graduate courses offered by other universities is satisfactory | | |  |
|  | 13 | | | Advertisement of post-graduate grant and scholarship opportunities is satisfactory | | |  |
|  | 14 | | | Management of post-graduate grants and scholarships is satisfactory | | |  |
|  | 15 | | | Opportunities for post-graduate students to pay exchange visits to other universities is satisfactory | | |  |
|  | 16 | | | Opportunities for post-graduate students to access courses at other universities is satisfactory | | |  |
|  | 17 | | | Support to career planning provided by the university/faculty/department is satisfactory | | |  |
|  | 18 | | | Career opportunities at the university is satisfactory | | |  |
| *The following questions relate to the role and function of mentors of post-graduate students* | | | | | | | |
| 87 | Does this university or faculty have a mentorship programme whereby post-graduate students are mentored (assisted) by academic staff during their course of training?   1. Yes 2. No [go to 99] 3. I don’t know [go to 99] | | | | | | |
| 88 | Have you been a formal mentor of any post-graduate students at this university?   1. Yes; how many?................ 2. No; why not?................[go to 99] | | | | | | |
| 89 | Do post-graduate students have a written action plan describing what to do and when during the course of post-graduate training?   1. Yes 2. No; why not?............ [go to 91] 3. I don’t know [go to 91] | | | | | | |
| 90 | Who developed this action plan in the post-graduate training courses that you have mentored? | | | | | | |
|  | 1 | | The post-graduate student | | | 1. Yes 2. No | |
|  | 2 | | The mentor(s) | | | 1. Yes 2. No | |
|  | 3 | | The department | | | 1. Yes 2. No | |
|  | 4 | | Others; specify whom:……………… | | | 1. Yes 2. No | |
| 91 | How well do you know the formal role of mentors in assisting post-graduate students?   1. Very well 2. Moderately well 3. Poorly [go to 93] | | | | | | |
| 92 | What are the sources of your knowledge about the formal role of mentors of post-graduate students? | | | | | | |
|  | 1 | | University or faculty handbook/guide | | | 1. Yes 2. No | |
|  | 2 | | Departmental handbook/guide | | | 1. Yes 2. No | |
|  | 3 | | University website | | | 1. Yes 2. No | |
|  | 4 | | Official briefing | | | 1. Yes 2. No | |
|  | 5 | | Informal discussions with university staff | | | 1. Yes 2. No | |
|  | 6 | | Informal discussions with post-graduate students | | | 1. Yes 2. No | |
|  | 7 | | Other; specify:……………………………. | | | 1. Yes 2. No | |
| 93 | How satisfied are you with your own competencies in mentoring post-graduate students?   1. Very satisfied 2. Moderately satisfied 3. Un-satisfied | | | | | | |
| 94 | Have you received any formal training in how to mentor post-graduate students?   1. Yes; where did you receive this training (name of institution and country)?...................[go to 96] 2. No | | | | | | |
| 95 | Would you be interested in receiving formal training in how to mentoring post-graduate students?   1. Yes 2. No; why not?................... | | | | | | |
| 96 | Are there any standards or rules for how much time you should spend on mentoring post-graduate students?   1. Yes; specify the amount of time in hours per week:………….. 2. No 3. I don’t know | | | | | | |
| 97 | How do you manage the time that you spend on mentoring post-graduate students? | | | | | | |
|  | 1 | | I keep specific “office opening hours” for mentoring students | | | 1. Yes 2. No | |
|  | 2 | | I offer mentorship based on appointments requested by the students | | | 1. Yes 2. No | |
|  | 3 | | I don’t; my office is always open for students when I am around | | | 1. Yes 2. No | |
|  | 4 | | Others; specify how:……………… | | | 1. Yes 2. No | |
| 98 | How satisfied are you with the regularity of your contacts with your post-graduate students?   1. Very satisfied 2. Moderately satisfied 3. Un-satisfied; specify why?......................... 4. I don’t know | | | | | | |
| *The following questions relate to post-graduate courses and teaching* | | | | | | | |
| 99 | Have you been involved in teaching post-graduate students at this faculty during the last couple of years?   1. Yes; specify in how many courses you taught:………………… 2. No; why not?.....................[go to 101] | | | | | | |
| 100 | How satisfied are/were you with teaching post-graduate students?   1. Very satisfied 2. Moderately satisfied 3. Un-satisfied; specify why?................... | | | | | | |
| 101 | How satisfied are you with the level of recognition provided by the university for teaching post-graduate students?   1. Very satisfied 2. Moderately satisfied 3. Un-satisfied; specify why?................... | | | | | | |
| 102 | How satisfied are you with your own teaching skills?   1. Very satisfied 2. Moderately satisfied 3. Un-satisfied | | | | | | |
| 103 | Have you received any formal training in how to teach?   1. Yes; where (name of training institution and country)…………..[go to 105] 2. No | | | | | | |
| 104 | Would you be interested in receiving formal training in how to teach?   1. Yes 2. No; why not?................... | | | | | | |
| 105 | Are there any compulsory courses for students enrolled in a post-graduate programme at this faculty or department?   1. Yes 2. No 3. I don’t know | | | | | | |
| 106 | Does this faculty or department offer a sufficiently comprehensive catalogue of post-graduate courses?   1. Yes [go to 108] 2. No 3. I don’t know [go to 108] | | | | | | |
| 107 | Which important courses do you find are missing?   1. ……………. 2. ……………. 3. ……………………………………………………… | | | | | | |
| 108 | Does the university, faculty or department encourage academic staff to develop new post-graduate courses?   1. Yes 2. No 3. I don’t know | | | | | | |
| 109 | Have you been involved in developing any new courses for post-graduate students within the last couple of years?   1. Yes 2. No; why not?....................... | | | | | | |
| 110 | Are there mechanisms in place at the university for reviewing the relevance and quality of courses offered to post-graduate students?   1. Yes 2. No [go to 115] 3. I don’t know [go to 115] | | | | | | |
| 111 | How satisfied are you with the effectiveness of these reviews?   1. Very satisfied 2. Moderately satisfied 3. Un-satisfied; specify why................... | | | | | | |
| 112 | How are the reviews carried out?   1. As internal reviews by the department(s) 2. As internal reviews by the faculty 3. As external reviews by consultancy firms or other training institutions 4. Other; specify how............. 5. I don’t know | | | | | | |
| 113 | How often are the reviews carried out?   1. About once every year 2. About once every two years 3. Other; specify how many years between each review?............. 4. I don’t know | | | | | | |
| 114 | Are post-graduate students involved in the evaluation of courses?   1. Yes 2. No 3. I don’t know | | | | | | |
| 115 | If you have suggestions, which have not been addressed elsewhere in this questionnaire, for improving the post-graduate programmes at this university, then please list them here: | | | | | | |
| **D. RESEARCH MANAGEMENT** | | | | | | | |
| 116 | Have you been actively involved in conducting research during the last couple of years?   1. Yes 2. No; why not?…………………[go to 133] | | | | | | |
| 117 | Are there any requirements on how much time you should spend on conducting research?   1. Yes; specify the proportion of time you should spend on research:………….. 2. No [go to 119] 3. I don’t know [go to 119] | | | | | | |
| 118 | How satisfied are you with the amount of time that you are expected to spend on conducting research?   1. Very satisfied 2. Moderately satisfied 3. Un-satisfied; specify why................... | | | | | | |
| 119 | Is your performance as a researcher assessed by the university, faculty or department?   1. Yes; how often?.................... 2. No [go to 123] 3. I don’t know [go to 123] | | | | | | |
| 120 | What are the main criteria used by the university to assess your research performance? | | | | | | |
|  | 1 | | Published papers in international scientific peer reviewed journals | | | 1. Yes 2. No | |
|  | 2 | | Published papers in any journals | | | 1. Yes 2. No | |
|  | 3 | | Impact factors of journals in which papers are published | | | 1. Yes 2. No | |
|  | 4 | | Dissemination of research findings to the public or to politicians | | | 1. Yes 2. No | |
|  | 5 | | Research funding attracted | | | 1. Yes 2. No | |
|  | 6 | | Other; specify:……………. | | | 1. Yes 2. No | |
|  | 7 | | I don’t know | | | 1. Yes 2. No | |
| 121 | How satisfied are you with the fact that your performance as a researcher is being assessed?   1. Very satisfied 2. Moderately satisfied 3. Un-satisfied; specify why................... | | | | | | |
| 122 | How satisfied are you with the method used to assess your research performance?   1. Very satisfied 2. Moderately satisfied 3. Un-satisfied; specify why................... | | | | | | |
| 123 | Are you informed about the outcome of the assessment of your performance as a researcher?   1. Yes 2. No | | | | | | |
| 124 | How satisfied are you with your own research skills?   1. Very satisfied 2. Moderately satisfied 3. Un-satisfied; specify why................... | | | | | | |
| 125 | Have you received any formal training in research methodology?   1. Yes; where (name of training institution and country)…………..[go to 127] 2. No | | | | | | |
| 126 | Would you be interested in receiving formal training in research methodology?   1. Yes 2. No; why not?................... | | | | | | |
| 127 | Have you published any papers in international scientific peer reviewed journals during the last two years?   1. Yes; how many?................. 2. No; why not?......................... | | | | | | |
| 128 | Have you ever published any papers in international scientific peer reviewed journals?   1. Yes; how many?................. 2. No; why not?......................... | | | | | | |
| 129 | Please express your opinion from your position as a researcher about the facilities and services (items 1-11) at this university using the following scale from 1-4 (or 5 if you don’t know):  1. Strongly agree 2. Agree 3. Disagree 4. Strongly disagree 5. I don’t know  Answer ↓ | | | | | | |
|  | 1 | | Internet accessibility is satisfactory | | | |  |
|  | 2 | | Library facilities is satisfactory | | | |  |
|  | 3 | | Opportunities to share libraries between departments/faculties is satisfactory | | | |  |
|  | 4 | | Opportunities to share libraries between institutions is satisfactory | | | |  |
|  | 5 | | Laboratories are adequately equipped | | | |  |
|  | 6 | | Laboratories are adequately staffed | | | |  |
|  | 7 | | Opportunities to share laboratories between departments/faculties is satisfactory | | | |  |
|  | 8 | | Opportunities to share laboratories between institutions is satisfactory | | | |  |
|  | 9 | | Support provided to pay exchange visits to other universities is satisfactory | | | |  |
|  | 10 | | Support provided to participate in international conferences or meetings is satisfactory | | | |  |
|  | 11 | | Support provided to participate in courses at other universities is satisfactory | | | |  |
|  | 12 | | Career opportunities at the university is satisfactory | | | |  |
| 130 | Have you paid any exchange visits to other (local or international) research institutions during the last two years?   1. Yes; how many?................. 2. No; why not?......................... [go to 132] | | | | | | |
| 131 | Where did funding for the exchange visits come from? | | | | | | |
|  | 1 | The university, faculty or department | | | | 1. Yes 2. No | |
|  | 2 | The government | | | | 1. Yes 2. No | |
|  | 3 | A local partner institution | | | | 1. Yes 2. No | |
|  | 4 | An international partner institution | | | | 1. Yes 2. No | |
|  | 5 | A global organisation or funding agency | | | | 1. Yes 2. No | |
|  | 6 | Other; specify…………… | | | | 1. Yes 2. No | |
|  | 7 | I don’t know | | | | 1. Yes 2. No | |
| 132 | Have you participated in any international conferences or meetings during the last two years?   1. Yes; how many?................. 2. No; why not?......................... [go to 134] | | | | | | |
| 133 | Where did funding for your participation in international conferences or meetings come from? | | | | | | |
|  | 1 | The university, faculty or department | | | | 1. Yes 2. No | |
|  | 2 | The government | | | | 1. Yes 2. No | |
|  | 3 | A local partner institution | | | | 1. Yes 2. No | |
|  | 4 | An international partner institution | | | | 1. Yes 2. No | |
|  | 5 | A global organisation or funding agency | | | | 1. Yes 2. No | |
|  | 6 | Other; specify…………… | | | | 1. Yes 2. No | |
|  | 7 | I don’t know | | | | 1. Yes 2. No | |
| 134 | Have you taken any courses during the last two years for the purpose of upgrading your personal technical or scientific knowledge and skills?   1. Yes; how many?................. 2. No; why not?......................... [go to 136] | | | | | | |
| 135 | Where did funding for your course participation come from? | | | | | | |
|  | 1 | | The university, faculty or department | | | 1. Yes 2. No | |
|  | 2 | | The government | | | 1. Yes 2. No | |
|  | 3 | | A local partner institution | | | 1. Yes 2. No | |
|  | 4 | | An international partner institution | | | 1. Yes 2. No | |
|  | 5 | | A global organisation or funding agency | | | 1. Yes 2. No | |
|  | 6 | | Other; specify…………… | | | 1. Yes 2. No | |
|  | 7 | | I don’t know | | | 1. Yes 2. No | |
| 136 | Have you received any formal training in research ethics?   1. Yes; where (name of training institution and country)…………..[go to 138] 2. No; why not?......................... | | | | | | |
| 137 | Would you be interested in receiving formal training in research ethics?   1. Yes 2. No; why not?................... | | | | | | |
| 138 | Have you received any formal training in how to write scientific publications?   1. Yes; where (name of training institution and country)…………..[go to 140] 2. No | | | | | | |
| 139 | Would you be interested in receiving formal training in how to write scientific publications?   1. Yes 2. No; why not?................... | | | | | | |
| 140 | Have you received any formal training in how to attract research funding?   1. Yes; where (name of training institution and country)…………..[go to 142] 2. No | | | | | | |
| 141 | Would you be interested in receiving formal training in how to attract research funding?   1. Yes 2. No; why not?................... | | | | | | |
| 142 | If you have suggestions, which have not been addressed elsewhere in this questionnaire, for improving the research environment at this university, then please list them here: | | | | | | |
