# Supplementary material for: Needs assessment to strengthen capacity in water and sanitation research in Africa: experiences of the African SNOWS consortium
Source: Health Res Policy Syst. 2014 Dec 15;12:68. doi: 10.1186/1478-4505-12-68 (PMC4274706; doi:10.1186/1478-4505-12-68)
Supplement: Supplementary file 1 — Additional file 1: Zip file containing questionnaires used in the needs assessment. (ZIP 338 KB) [file 12961_2014_366_MOESM1_ESM.zip › SNOWS.Mapping. Annex.2.Template for mapping of scientific resources at Department level.13.2.2010.doc]

**Mapping of competences and activities regarding research and research training (supervision, induction, tuition, course work etc related to post graduate and doctorate programmes ) related to Water, Sanitation and Environmental Health at the University of XXX 2005-2010.**

**Use one form for each Department engaged in research and research training related to Water, Sanitation and Environmental Health**

**Contact Information:**

Name of Department:

Under which College or Faculty:

Contact person at Department:

Phone/contact person:

E-mail/contact person:

**A. Description of research:**

A.1. Description of research areas in one sentence pr area:

A.2. Scientific focus (1 page maximum):

A.3 Current research schools and/or groups related to areas covered by SNOWS:

A.4 Current and Completed Research projects (mark the ongoing research projects with a star *):

**B. Scientific staff, PhD fellows and post graduate students:**

B.1 Number of scientific staff (Assistant Professors, Associate Professors, Professors, Lecturers, other..)

| ***Category*** | ***No. of Scientific Staff*** | ***No of CVs attached*** |
| --- | --- | --- |
| *Professor* |  |  |
| *Assoc. Professor* |  |  |
| *Assist. Professor* |  |  |
| *Lecturer* |  |  |
| *Other Scientific staff* |  |  |
| *Total* |  |  |

The table above registers only current permanent and project funded staff, and does thus not include former staff members in the period under consideration.

B.2 Number of PhD fellows currently attached to the Department**:**

Of these how many are engaged in SNOWS relevant research:

B.3 Number of post graduate students (MA/MSc/Mphil or other) currently attached to the

Department**:**

Of these how many are engaged in SNOWS relevant research:

**C. Funding:**

C.1 External research funds (only those funds related to the responsibilities of the Department):

*2005–2010: xxx,xxx,xxx.xx [local currency]	equivalent to USD:xxx,xxx.xx*

C.2. How are PhDs at the department funded:

\# PhD students self-funded:

\# PhD students funded by funds available to the department:

\# PhD students funded by national institutions:

\# PhD students funded by external funds:

**D. Publications:**

D.1 Number of peer-reviewed articles in international scientific journals (2005–2010):

D.2 Number of conference abstracts published in conference proceedings:

D.3 No. of articles in national journals:

D.4 Major reports:

**E. Research networks:**

Give brief descriptions of the departments research networks relevant to SNOWS with:

International universities and research institutions (eg.CGIAR)

National universities

Sector Ministries, Departments and Agencies

National NGOs and CBOs

International NGOS

Private sector operators

International development partners (Banks, multilateral and bilateral Donors)

Others

-----------

Use the cells below for each network. Add additional cells if needed.

E.1

E.2

E.3

**F. Research Training:**

F.1. Titles of courses at post graduate level (relevant to Water, Sanitation and Environmental Health) with contribution from the Department:

1. [Name of course], [Name and department of course manager]
2. [Name of course], [Name and department of course manager]
3. [Name of course], [Name and department of course manager]

…add more if needed…

F.2. Titles of courses and seminars at Doctorate level (relevant to Water, Sanitation and Environmental) with contribution from the Department:

1. [Name of course], [Name and Department of course manager]
2. [Name of course], [Name and Department of course manager]
3. [Name of course], [Name and Department of course manager]

…add more if needed…

**Appendix 1: Publications**

*The list covers peer-reviewed publications with department contribution within the period 2005–2010:.*

1. **: …**
2. **: …**

**Appendix 2. Projects**. The list includes larger research projects initiated, implemented and terminated in the period 2005-2010

| **Project responsible party / Principal Investigator** | **Funding (USD)** | **External**  **(yes: X)** | **Project title** | **Start year –**  **end year** |
| --- | --- | --- | --- | --- |
|  |  |  |  |  |
|  |  |  |  |  |
| Add more rows to the table if needed |  |  |  |  |
| **Total** |  |  |  |  |
