# Supplementary material for: Needs assessment to strengthen capacity in water and sanitation research in Africa: experiences of the African SNOWS consortium
Source: Health Res Policy Syst. 2014 Dec 15;12:68. doi: 10.1186/1478-4505-12-68 (PMC4274706; doi:10.1186/1478-4505-12-68)
Supplement: Supplementary file 1 — Additional file 1: Zip file containing questionnaires used in the needs assessment. (ZIP 338 KB) [file 12961_2014_366_MOESM1_ESM.zip › SNOWS.Mapping.Annex.3.Template for List of Documents.doc]

**Annex 3**

**List of Documents**

**- Mapping of Research Capacity African Partners of SNOWS**

|  | Insert the title of relevant documents in this column:  Insert more rows if needed | Insert the contact details (name, email) of the person from whom the document can be obtained. | Insert x if available as electronic document | Insert hyperlink if the document is available on the internet/ university website. |
| --- | --- | --- | --- | --- |
| **1** | **Research Management** |  |  |  |
| 1.1 | **Organisation** |  |  |  |
| 1.1.1 | ***Organisational structure*** |  |  |  |
|  | Document 1… | Name and email.: | (x) | www…. |
|  | Document 2… |  |  |  |
|  | Add more rows if needed… |  |  |  |
| 1.1.2 | ***Policies and strategies*** |  |  |  |
|  | Document 1… |  |  |  |
|  | Add more rows if needed… |  |  |  |
| 1.1.3 | ***Funding of Research*** |  |  |  |
|  | Document 1… |  |  |  |
| 1.1.4 | ***Financial Management*** |  |  |  |
|  | Document 1… |  |  |  |
| 1.2 | **Resources** |  |  |  |
|  | Document 1… |  |  |  |
| 1.2.1 | ***Departments of relevance to SNOWS*** |  |  |  |
|  | Document 1… |  |  |  |
| 1.2.2 | ***Staff strength*** |  |  |  |
|  | Document 1… |  |  |  |
| 1.2.3 | ***Continued professional development and staff mobility*** |  |  |  |
|  | Document 1… |  |  |  |
| 1.2.4 | ***The Research Infrastructure:*** |  |  |  |
|  | Document 1… |  |  |  |
| 1.2.5 | ***Study Sites and External Networks*** |  |  |  |
|  | Document 1… |  |  |  |
| 2 | **PhD Programme** |  |  |  |
| 2.1 | **Organisation** |  |  |  |
| 2.1.1 | ***Organisational structure*** |  |  |  |
|  | Document 1… |  |  |  |
| 2.1.2 | ***Policies and strategies*** |  |  |  |
|  | Document 1… |  |  |  |
| 2.1.3 | ***Rules and Regulations for the PhD programme*** |  |  |  |
|  | Document 1… |  |  |  |
| 2.1.4 | ***Funding of PhD Research*** |  |  |  |
|  | Document 1… |  |  |  |
| 2.1.5 | ***Monitoring and evaluation*** |  |  |  |
|  |  |  |  |  |
| **2.2** | **Training & Research environment / infrastructure** |  |  |  |
| 2.2.1 | ***Training – development of research and other skills*** |  |  |  |
|  | Document 1… |  |  |  |
| 2.2.2 | ***Supervision of PhDs*** |  |  |  |
|  | Document 1… |  |  |  |
| 2.2.3 | ***Technical support staff/lab technicians / librarians /IT support*** |  |  |  |
|  | Document 1… |  |  |  |
| 2.2.4 | ***Administrative Staff Strength*** |  |  |  |
|  | Document 1… |  |  |  |
|  |  |  |  |  |
| 2.3 | **PhD fellows** |  |  |  |
| 2.3.1 | ***PhD Fellows in areas relevant to SNOWS*** |  |  |  |
|  | Document 1… |  |  |  |
| 2.3.2 | ***Mobility of PhD fellows*** |  |  |  |
|  | Document 1… |  |  |  |
| 2.3.3 | ***PhD admission*** |  |  |  |
|  | Document 1… |  |  |  |
| 2.3.4 | ***Monitoring progress*** |  |  |  |
|  | Document 1… |  |  |  |
| 2..3.5 | ***Research Budget*** |  |  |  |
|  | Document 1… |  |  |  |
| 2.3.6 | ***Conflict resolution*** |  |  |  |
|  | Document 1… |  |  |  |
| 2.3.7 | ***Assessment of the PhD fellow / dissertation and defence*** |  |  |  |
|  | Document 1… |  |  |  |
| 2.3.8 | ***Induction / support for young lecturers (PhD fellows required to teach/lecture)*** |  |  |  |
|  | Document 1… |  |  |  |
| 2.3.9 | ***Student Networks and Representation*** |  |  |  |
|  | Document 1… |  |  |  |
| 2.3.10 | ***PhD Newsletter or Bulletin*** |  |  |  |
|  | Document 1… |  |  |  |
|  |  |  |  |  |
| 3 | **Post Graduate Programmes** |  |  |  |
| 3.1 | **Organisation** |  |  |  |
| 3.1.1 | ***Organisational structure*** |  |  |  |
|  | Document 1… |  |  |  |
| 3.1.2 | ***Policies and strategies*** |  |  |  |
|  | Document 1… |  |  |  |
| 3.1.3 | ***Rules and Regulations for the post graduate programme*** |  |  |  |
|  | Document 1… |  |  |  |
| 3.1.4 | ***Funding of post graduate research*** |  |  |  |
|  | Document 1… |  |  |  |
| 3.1.5 | ***Monitoring and evaluation*** |  |  |  |
|  | Document 1… |  |  |  |
| **3.2** | **Training & Research environment / infrastructure** |  |  |  |
|  |  |  |  |  |
| 3.2.1 | ***Training – development of research and other skills*** |  |  |  |
|  | Document 1… |  |  |  |
| 3.2.2 | ***Tuition of post graduate students*** |  |  |  |
|  | Document 1… |  |  |  |
| 3.2.3 | ***Technical support staff/lab technicians / librarians /IT support*** |  |  |  |
|  | Document 1… |  |  |  |
| 3.2.4 | ***Administrative Staff Strength*** |  |  |  |
|  | Document 1… |  |  |  |
|  |  |  |  |  |
| 3.3 | **Post graduate students** |  |  |  |
| 3.3.1 | ***Post graduate students in areas relevant to SNOWS*** |  |  |  |
|  | Document 1… |  |  |  |
| 3.3.2 | ***Mobility of Post Graduate students*** |  |  |  |
|  | Document 1… |  |  |  |
| 3.3.3 | ***Post Graduate admission*** |  |  |  |
|  | Document 1… |  |  |  |
| 3.3.4 | ***Monitoring progress*** |  |  |  |
|  | Document 1… |  |  |  |
| 3. 3.5 | ***Research Budget*** |  |  |  |
|  | Document 1… |  |  |  |
| 3.3.6 | ***Conflict resolution*** |  |  |  |
|  | Document 1… |  |  |  |
| 3.3.7 | ***Assessment of the Post Graduate students / thesis*** |  |  |  |
|  | Document 1… |  |  |  |
| 3.3.8 | ***Student Networks and Representation*** |  |  |  |
|  | Document 1… |  |  |  |
| 3.3.9 | ***Newsletter or Bulletin for Post Graduate students*** |  |  |  |
|  | Document 1… |  |  |  |
|  |  |  |  |  |
