# Supplementary material for: Needs assessment to strengthen capacity in water and sanitation research in Africa: experiences of the African SNOWS consortium
Source: Health Res Policy Syst. 2014 Dec 15;12:68. doi: 10.1186/1478-4505-12-68 (PMC4274706; doi:10.1186/1478-4505-12-68)
Supplement: Supplementary file 1 — Additional file 1: Zip file containing questionnaires used in the needs assessment. (ZIP 338 KB) [file 12961_2014_366_MOESM1_ESM.zip › SNOWS.Mapping.Annex.5.CV-format,PhD-fellows.11.2.2010.doc]

**University of XXX**

**CV – PhD Fellow Profile**

| Name |  | |
| --- | --- | --- |
| Department |  | |
| Working address |  | |
| Contact details | Email, main:  Email, secondary:  Phone (office): | Skype:  Phone (Cell): |
| Formal education |  | |
| Current position |  | |
| Titel of PhD Project |  | |
| Year of Enrolment – expected year of PhD Defence | 20xx – 20xx | |
| Description of PhD project (max 5 lines) |  | |
| Name and Department of main supervisor |  | |
| Contact details of main supervisor | Email, main:  Email, secondary:  Phone (office): | Skype:  Phone (cell): |
| Name and address of co-supervisor |  | |
| Contact details of co-supervisor | Email, main:  Email, secondary:  Phone (office): | Skype:  Phone (cell): |
| Name and address of additional co-supervisor (if relevant) |  | |
| Contact details of project supervisor | Email, main:  Email, secondary:  Phone (office): | Skype:  Phone (cell): |
| Scientific Production | Number of peer-reviewed articles in international scientific journals: | |
|  | Number of conference abstracts published in conference proceedings: | |
|  | No. of articles in national journals: | |
|  | Major reports: | |
| List max. 5 selected articles related to SNOWS |  | |
