# Supplementary material for: Needs assessment to strengthen capacity in water and sanitation research in Africa: experiences of the African SNOWS consortium
Source: Health Res Policy Syst. 2014 Dec 15;12:68. doi: 10.1186/1478-4505-12-68 (PMC4274706; doi:10.1186/1478-4505-12-68)
Supplement: Supplementary file 1 — Additional file 1: Zip file containing questionnaires used in the needs assessment. (ZIP 338 KB) [file 12961_2014_366_MOESM1_ESM.zip › SNOWS.mapping.Annex4.CV-format.scientific staff profiles. 11.2.2010.doc]

**University of XXX**

**CV - Scientific Staff Profile**

| Name |  | |
| --- | --- | --- |
| Department |  | |
| Working address |  | |
| Contact details | Email, main:  Email, secondary: | Skype: |
|  | Phone (office): | Phone (Cell): |
| Formal education |  | |
| Current position |  | |
| Research area and expertise of relevance for SNOWS (max 5 lines) |  | |
| Current research project(s) relevant to SNOWS | 1. [Title of research project], [Responsibility in team]  2. [Title of research project], [Responsibility in team]  ..add more if needed.. | |
| Courses at Post Graduate level, relevant to SNOWS | 1. [Name of course], [Responsibility in course] 2. [Name of course], [Responsibility in course]   ..add more if needed.. | |
| Courses and seminars at Doctorate level relevant to SNOWS | 1. [Name of course], [Responsibility in course] 2. [Name of course], [Responsibility in course]   ..add more if needed.. | |
| Other courses of relevance to SNOWS |  | |
| Number of Post Graduate students tutored at present |  | |
| Number of PhDs supervised at present |  | |
| Number of PhD fellows supervised that are/were not enrolled at own department (last 5 years) | Number:  Enrolled at :  1:  2:  .. | |
| Scientific Production | Number of peer-reviewed articles in international scientific journals: | |
|  | Number of conference abstracts published in conference proceedings: | |
|  | No. of articles in national journals: | |
|  | Major reports: | |
| Distinctions |  | |
| List max. 10 selected articles related to SNOWS |  | |
