# Supplementary material for: Needs assessment to strengthen capacity in water and sanitation research in Africa: experiences of the African SNOWS consortium
Source: Health Res Policy Syst. 2014 Dec 15;12:68. doi: 10.1186/1478-4505-12-68 (PMC4274706; doi:10.1186/1478-4505-12-68)
Supplement: Supplementary file 1 — Additional file 1: Zip file containing questionnaires used in the needs assessment. (ZIP 338 KB) [file 12961_2014_366_MOESM1_ESM.zip › SNOWS-Mapping.Annex.1.Guidelines for reporting on Mapping.rev-1.15.2.2010.doc]

**Annex 1**

**Guidelines for**

**Mapping of Research Capacity**

**African Partners of SNOWS**

**Name of SNOWS partner university: ________________**

| **Name of Water and Sanitation group members:** | **Contact details:** |  |
| --- | --- | --- |
| **Name:** | **Phone (cell):** | **e-mail** |
|  | **Phone (Office):** | **Skype:** |
|  | | |
| **Name:** | **Phone (cell):** | **e-mail** |
|  | **Phone (Office):** | **Skype:** |
|  | | |
| **Name:** | **Phone (cell):** | **e-mail** |
|  | **Phone (Office):** | **Skype:** |
|  | | |
| **Name:** | **Phone (cell):** | **e-mail** |
|  | **Phone (Office):** | **Skype:** |
|  | | |
| **Name:** | **Phone (cell):** | **e-mail** |
|  | **Phone (Office):** | **Skype:** |
|  | | |
| **Name:** | **Phone (cell):** | **e-mail** |
|  | **Phone (Office):** | **Skype:** |

Add more names if needed…

|  | *The points in this column are the main points to address in the mapping report. Points refer to headings and subheadings of* ***Report Format for Mapping***  *A brief instruction is provided to guide Water & Sanitation groups on key issues to cover under each heading.* | *In this column, more detailed guidelines are given for some of the complex areas. Cover the questions to the extent possible. If there are areas that cannot be covered due to confidentiality, it would be helpful if a note is made on this in the Mapping Report under the relevant section.* | *Use this column for notes by the Water and Sanitation group on which Office/colleagues should provide the information and which Water & Sanitation group member should be responsible for ensuring that feedback is delivered timely and to satisfactory quality.* |
| --- | --- | --- | --- |
| **1** | **Research Management** |  |  |
| 1.1 | **Organisation** |  |  |
| 1.1.1 | ***Organisational structure*** |  |  |
|  | Summarise the organisational structure regarding research & research management at your university and provide information on relevant documents and hyperlinks. | a) Who has responsibility for general oversight of research and research management at the university? b) What are the core tasks for this position? c) Describe the organisational structure of the university regarding research & research management and include organisational chart, with core tasks of key stakeholders in the chart (i.e. from the level of Vice chancellor Dean, college, faculty, school, department, and sub-department levels - where relevant ) d) Provide list of committees, boards and directorships that have role in Research & Research Management and describe the role / terms of reference of each entity and the relationship between them. e) List relevant documents describing organisation structure using the *Template for List of Documents* provided with this mapping tool. |  |
|  |  |  |  |
| 1.1.2 | ***Policies and strategies*** |  |  |
|  | Describe and provide list of policies, strategies and action plans on research and research management. If specific policies, strategies and action plans exist for research and research management for Water, Sanitation and Environmental Health these should described too. | a) List relevant policy, strategy and action plan documents using the *Template for List of Documents* provided with this mapping tool. Distinguish between which institutional level is responsible for the strategy: University/College/Faculty/Department. Include also strategies & guidelines (or standard operating procedures - SOPs) for interdisciplinary and interdepartmental research and training (eg. Between engineering, epidemiology, environmental microbiology, and behavioural sciences); policies on completion and completion rates. b) Describe and point out which policies, strategies and action plans are relevant and why? |  |
| 1.1.3 | ***Funding of Research*** |  |  |
|  | Summarise the organisational structure of the university related to fund raising | a) Does your university have a separate fund raising office(s)? b) If so: a) Which organisational levels do have such office(s)? c) Describe the mandate of the office(s) and repartition of responsibilities with other units/staff.. d) If not: Who keeps general oversight of fund raising for research? e) Has this system for support to fundraising been evaluated recently? f) List relevant documents using the *Template for List of Documents* |  |
|  | Summarise the policies, strategies and action plans for funding of research projects? | a) List relevant documents using *Template for List of Documents.* |  |
|  | Have policies/strategies on external funding been evaluated recently? | a) Summarise the findings and recommendations of this evaluation. b) List relevant documents using *Template for List of Documents.* |  |
|  | Provide documentation on key features on funding of research related to water, sanitation and environmental health. | a) External/national sources of research funds for research in areas relevant to SNOWS the last five years, including figures if possible. b) External/international sources of research funds for research in areas relevant to SNOWS the last five years, including figures if possible. c) Funding from own resources for research in areas relevant to SNOWS the last five years, including figures if possible |  |
|  |  |  |  |
| 1.1.4 | ***Financial Management*** |  |  |
|  | Describe the organisational structure related to management of research funds. | a) Does your university have a separate office responsible for management of research funds? b) If so: Which organisational levels do have such office(s)? c) Describe the mandate of the office(s) and repartition of responsibilities with other units and with project coordinators/managers. E.g. can external research funds be managed at department level? |  |
|  | Summarise university rules and regulations related to management of research funds. | a) Does your university have standard rules and regulation, financial guidelines or codes of practice for managing research funds? b) Has these rules and regulations & guidelines been evaluated recently? a) Provide list of documents relevant to management of research funds using the *Template for List of Documents*. |  |
|  | Describe your management information system (MIS) related to managing research funds? | a) Summarise the key features of your MIS. b) Describe in more detail the repartition of the responsibilities of the research project manager and the financial manager/accountant in financial monitoring and accounting/reporting. c) Describe in more detail the repartition of the responsibilities of the research project manager and the financial manager/accountant in overall project monitoring and reporting. d) Provide list of documents relevant to management of research funds using the *Template for List of Documents*. |  |
|  | Describe the role of the internal auditor with regard to external research funds, e.g. are external research funds audited by internal or external auditors. |  |  |
|  |  |  |  |
| 1.2 | **Resources** |  |  |
| 1.2.1 | ***Departments of relevance to SNOWS*** |  |  |
|  | List the various departments with interest in and relevance for SNOWS and describe the research areas covered by each department | Let each department fill in the *Template for Mapping of Scientific Resources at Department Level* and extract information from section A.1 of each filled in form. All department forms should be annexed to the Mapping Report. |  |
|  |  |  |  |
| 1.2.2 | ***Staff strength*** |  |  |
|  | Summarise ***scientific staff strength*** of relevance for research and research management within areas covered by SNOWS. | Assign to each department the responsibility of providing staff profiles of scientific staff covering areas of relevance to SNOWS. Use the *Template for Mapping of Scientific Resources at Department Level* and *CV Format –Scientific staff profiles*  *Include the filled in templates and staff profiles in annex to the mapping report.* |  |
|  | How is academic staff performance assessed? | Summarise mechanism for staff assessment including whether there are set targets for each researcher/academic staff for the various tasks assigned. |  |
|  | What are the practical implications of staff assessment? | E.g. are incentives provided to successful staff? |  |
|  |  |  |  |
| 1.2.3 | ***Continued professional development and staff mobility*** |  |  |
|  | Describe staff training and continued education opportunities, | a. …including exchange and staff mobility programme arrangements offered to scientific staff – b. Is any training compulsory? c. Describe arrangements for mentorship of staff, if available. |  |
|  |  |  |  |
| 1.2.4 | ***The Research Infrastructure:*** |  |  |
|  | Describe the research infrastructure at the university and policies, strategies and action plans addressing these areas. Summarise the findings and recommendations if the infrastructure has been evaluated. | a) Summarise the policies and strategies and provide the documentation in the *Template for list of documents.* b) Describe access to library support for scientific staff, post graduate students and PhD fellows. c) Describe the access to electronic journals and up to date text books for scientific staff, post graduate students and PhD fellows d) Describe the availability of laboratory space, equipment and lab technicians for scientific staff, post graduate students and PhD fellows e) Describe the internet access and computer availability for scientific staff, post graduate students and doctorate fellows. f) Describe other relevant aspects of the research infrastructure. g) Summarise evaluation reports. |  |
|  |  |  |  |
| 1.2.5 | ***Study Sites and External Networks*** |  |  |
|  | Identify whether the University has particular study sites relevant to SNOWS developed over several years. Describe your findings. |  |  |
|  | Describe existing networks and strengths in research collaboration with external partners which offer opportunities for SNOWS? | Extract the information from *Template for Mapping of Scientific Resources at Department Level* when departments have filled in the templates.  Include brief descriptions of research networks with:  International universities and research institutions (eg.CGIAR)  National universities  Sector Ministries, Departments and Agencies  National NGOs and CBOs  International NGOS  Private sector operators  International development partners (Banks, multilateral and bilateral Donors)  Others |  |
|  |  |  |  |
| 2 | **PhD Programme** |  |  |
| 2.1 | **Organisation** |  |  |
| 2.1.1 | ***Organisational structure*** |  |  |
|  | Summarise the organisational structure of the PhD Programme at the university and provide information on relevant documents and hyperlinks. | a) Who has responsibility for general oversight of doctorate programmes at the university? b) What are the core tasks for this position? c) Describe the organisational structure of the university regarding doctorate programme(s) and include organisational chart, with core tasks of key stakeholders in the chart (i.e. from the level of Vice Chancellor, Dean, college, faculty, school, department, and sub-department levels - where relevant ) d) Provide list of committees, boards and directorships that have role in the doctorate programmes and describe the role / terms of reference of each entity and the relationship between them. e) List relevant documents describing organisation structure using the *Template for List of Documents* provided with this mapping tool. |  |
| 2.1.2 | ***Policies and strategies*** |  |  |
|  | Describe and provide list of policies, strategies and action plans on doctorate programmes. If particular policies, strategies and action plans exist for doctorate programmes in the areas of Water, Sanitation and Environmental Health, these should described too. | a) List relevant policy, strategy and action plan documents using the *Template for List of Documents* provided with this mapping tool. Distinguish between which institutional level is responsible for the strategy: University/College/Faculty/Department. Include also strategies & guidelines (or standard operating procedures - SOPs) for interdisciplinary and interdepartmental research and training (eg. Between engineering, epidemiology, environmental microbiology, and behavioural sciences); policies on completion and completion rates. b) Describe and point out which policies, strategies and action plans are relevant and why? |  |
| 2.1.3 | ***Rules and Regulations for the PhD programme*** |  |  |
|  | Summarise and provide list of your institutional regulations for doctorate programmes. | a) Summarise the rules & regulations governing the doctorate programmes. Specify which body is responsible for decisions regarding the rules & regulations (incl. codes of practice) and who or which body is responsible for ensuring compliance with the rules and regulations. b) List the relevant rules and regulations using the *Template for List of Documents* Regulations should include at least those concerning admission criteria, enrolment, supervision & joint supervision, induction, progress reporting, assessment criteria and mechanisms, format for thesis/dissertation, examination/defence, awarding of degree. Rules and regulations for PhD Fellow complaints and appeals processes, should be included if in place / available. c) How are rules and regulations related to PhD programmes communicated to PhD fellows and staff, e.g. is there a PhD handbook available for students and staff? Provide information on such handbook in the *Template for List of Documents* d) How are rules and regulations evaluated and adjusted/revised? Describe the decision making structure. |  |
|  |  |  |  |
| 2.1.4 | ***Funding of PhD Research*** |  |  |
|  | Describe the policies and strategies for funding of PhD research. Include current status of funding of PhD projects. | Some information may be collected from central level while other information required may be extracted from *Template for Mapping of Scientific Resources at Department Level* when departments have filled in the templates.   a) What are the policies and strategies for funding of PhD research projects? 1. Self financed PhDs + 2. own funding of PhDs by departments? 3. in-country funding of PhDs? 4. external funding of PhDs? b) Have policies/strategies on external funding of PhD projects been evaluated recently? c) Provide summary of fee structure for doctorate programmes and list relevant documents d) Provide, if possible, total external funding of doctorate research relevant to SNOWS and sources of funding. e) Total number of current PhD fellows at departments relevant to SNOWS:   # PhD students currently enrolled at the departments  # PhD students self-funded  # PhD students funded by funds available to the department:  # PhD students funded by national institutions  # PhD students funded by external funds   f) List relevant documents using *Template for List of Documents*. Include Financial Guidelines for PhD programmes if available |  |
|  |  |  |  |
| 2.1.5 | ***Monitoring and evaluation*** |  |  |
|  | Describe how the doctorate programme is monitored and evaluated, include the different administrative levels e.i. university/faculty/department and others if relevant. | a) How and when is/are doctorate programme(s) monitored and evaluated. b) How and when are policies and strategies monitored and evaluated. c) How and when are Rules and Regulations monitored and evaluated. d) List available monitoring and evaluation documents in Template for List of Documents e) How and to whom are monitoring and evaluation reports communicated? f) When the programme(s) were last evaluated. g) What formal opportunities are there for parties (PhD students, supervisors, others) to provide feedback (e.g. on experiences with the current system; suggestions for change; etc)? h) How is this feedback analysed and followed up? |  |
|  |  |  |  |
| **2.2** | **Training & Research environment / infrastructure** |  |  |
|  |  |  |  |
| 2.2.1 | ***Training – development of research and other skills*** |  |  |
|  | Summarise the framework provided by the university to facilitate PhD fellows’ learning processes. | Summarise which services and opportunities are offered to stimulate PhD fellows learning, and whether there is a policy on what weight are on which services, eg:  -Scientific courses  -Research methodology courses  -Generic courses on various aspects of personal/professional development  -Research or PhD schools and networks |  |
|  | What scientific courses are made available to PhD fellows in areas relevant to SNOWS. | List all relevant scientific courses offered to PhD fellows on research methodology. Include information on course manager and contact details for the manager. If course presentations are available on the internet, provide hyperlink.  Provide list of technical and scientific courses relevant to SNOWS at PhD level in relation to water supply, sanitation and environmental health (e.g. listed by faculty, department and theme) with key information on course duration, credits etc.  Course characteristics (e.g. objectives, mode of delivery, duration, timing, target groups, admission criteria and costing)  Provide in Annex a list courses offered to PhD fellows. It should be clear whether each course is particularly made for and delivered to PhD fellows (i.e.particularly targeted PhDs), or to other students, e.g post graduate students and open to PhDs. |  |
|  | How are PhD fellows expected to acquire knowledge on research methodologies | Are courses offered on research methodology? If so, are they strictly PhD courses or are the course targeted other academic levels, e.g. post graduate level?  Or are PhD fellows expected to acquire knowledge on research methodology through own studying and or through supervisor.  Or a mix of the above…  List all relevant courses offered to PhD fellows on research methodology. Include information on course manager and contact details for the manager. If course presentations are available on the internet, provide hyperlink.  The following topics are, among others, considered generic research methodology   - Philosophy of science and ethics of science. - Qualitative and quantitative research methodologies - Cross disciplinary research methodologies - Data processing and statistical analyses and use of statistical software - Research grant applications - Academic writing - Research communication |  |
|  | How are PhD fellows expected to acquire generic personal & professional skills? | List all relevant courses offered to PhD fellows on personal/professional development, e.g..  Personal development planning  Development of career/ employment skills?  Project management and budgeting  Effecting reading techniques  Presentation skills  IT skills and software (word, excel, powerpoint)  Career planning |  |
|  | Is it compulsory to take PhD courses? If so explain credit system |  |  |
|  | Does university have PhD level research schools and networks related to water supply, sanitation and environmental health? | Provide list of relevant research schools or networks, including documents describing each school/network or a URL address (deep link) if documents can be accessed on internet. |  |
|  |  |  |  |
| 2.2.2 | ***Supervision of PhDs*** |  |  |
|  | Describe the framework for supervision at the university | a) Explain whether there is an agreed generic terms of reference for supervisors or supervisors’ guidelines at the university outlining the role of the supervisor. b) How are supervisors appointed? What policies or guidelines exist to guide the process? c) Describe what training and / or mentoring (if any) is made available to new supervisors? d) How are supervisors evaluated, if at all? e) Is there any forum in which supervisor roles are discussed and experiences and lessons learned shared? f) Are there guidelines for joint supervision within the university? g) Are there guidelines for joint supervision from more than one institution? – including areas of responsibility; contract/agreement on supervision. Etc. h) Is there any provision of support for external supervisors, including honorarium, travel, per diem etc related? If so, does it cover both supervision and defence/evaluation? i) What is the expected turn around time for feedback from supervisors on drafts? |  |
|  |  |  |  |
| 2.2.3 | ***Technical support staff/lab technicians / librarians /IT support*** |  |  |
|  | Describe what services are available to PhD fellows from technical support staff. And summarise how support staff can keep updated in their professional areas. | a) Provide organisation chart showing which support staff are related to technical support of the PhD programmes, e.g. technical support staff / lab technicians / librarians / IT support, etc b) Describe opportunities for continuous professional development of technical support staff. |  |
|  |  |  |  |
| 2.2.4 | ***Administrative Staff Strength*** |  |  |
|  | Describe what admin staff are involved managing the PhD programmes and how admin staff can keep updated in their professional areas. | a) Provide organisation chart showing which support staff are related to administration of the PhD programmes. b) Describe opportunities for continuous professional development of administrative staff. |  |
|  |  |  |  |
|  |  |  |  |
| 2.3 | **PhD fellows** |  |  |
| 2.3.1 | ***PhD Fellows in areas relevant to SNOWS*** |  |  |
|  | List PhDs engaged in research relevant to SNOWS? | Provide consolidated list of PhDs involved in research relevant to SNOWS based on the *Template for Mapping of Scientific Resources at Department Level.*  Include PhD profiles in the annex to the mapping report using the  *CV format for PhD fellow profiles*. |  |
|  |  |  |  |
| 2.3.2 | ***Mobility of PhD fellows*** |  |  |
|  | Describe support provided and opportunities open for PhD fellows to follow courses or undertake part of the research abroad? | Explain system and funding, regulations etc. Are fellows merited for participation in external courses? |  |
|  |  |  |  |
| 2.3.3 | ***PhD admission*** |  |  |
|  | How are PhD positions advertised? |  |  |
|  | What are admission requirements? | List relevant documents using *Template for List of Documents* |  |
|  | May admission requirements be revised / relaxed if applicants do not comply? |  |  |
|  | What options are in place to bring candidates to the required level prior to or following enrolment? |  |  |
|  | Is an post gradtuate degree required for all applicants, including applicants with a degree in medicine? |  |  |
|  | What are the interviewing, recruitment, selection, and admission procedures? |  |  |
|  | Who monitors and enforces adherence to procedures? |  |  |
|  |  |  |  |
| 2.3.4 | ***Monitoring progress*** |  |  |
|  | Do all PhD fellows commit to a PhD plan or a contract between the PhD fellow and the supervisor(s)? | a) If so explain: When is the plan due to be made? b) Mechanism for approval of plan? c) Mechanism for revision/adjustment of plan? |  |
|  | What are the formal mechanisms for monitoring and supporting PhD fellow progress, including formal review stages/milestones? | a) How do students record their milestones and achievements? b) What is the supervisor’s role in monitoring progress? c) What are the roles of University/faculty/department administrators in monitoring student progress? d) If there is a formal PhD plan, who monitors adherence to the plan? e) If progress reports are made, are they made available to 1) the PhD Fellow, 2) all supervisors and 3) admin staff at department/faculty/postgraduate school/university level. f) Is there a manual and/or on-line tracking system for PhD fellows’ progress? g) What are the procedures for managing PhD fellows not performing to standards? |  |
|  | Do all PhD fellows develop a personal development plan | Are PhD fellows guided or offered training in developing and using a personal development plan |  |
|  | Do all PhD fellows develop a contract with their supervisors? | e.g. related to  -frequency of meetings with supervisors  -record keeping  - confidentiality  -milestones for the research project  -conflict resolution  - issues to do with supervisors reading of drafts/revisions. |  |
|  | What is the average time to complete a PhD from the departments involved in SNOWS? |  |  |
|  |  |  |  |
| 2..3.5 | ***Research Budget*** |  |  |
|  | Who is responsible for the PhD budget and decisions on the use of this budget, if a budget exist? |  |  |
|  |  |  |  |
| 2.3.6 | ***Conflict resolution*** |  |  |
|  | Is there a clear process to resolve possible conflicts between supervisors? |  |  |
|  | Is there a clear process to resolve possible conflicts between PhD fellow and supervisor? Can a supervisor be replaced? |  |  |
|  |  |  |  |
| 2.3.7 | ***Assessment of the PhD fellow / dissertation and defence*** |  |  |
|  | Describe the framework for assessment of PhD fellows. | a) Summarise the assessment criteria or guidelines for assessment of PhDs. b) When and how are the criteria made communicated to the student? c) Are there any informal assessment opportunities (e.g examples) available to PhD Fellows? d) How is the PhD assessment / defence committee identified? e) Are external examiners appointed? If so how? f) What is the set (if any) timeframe for providing the assessment result when students have handed in the dissertation? g) What procedures are in place if timeframe for assessment is not complied with? |  |
|  |  |  |  |
| 2.3.8 | ***Induction / support for young lecturers (PhD fellows required to teach/lecture)*** |  |  |
|  | Is induction and guidance provided to PhDs as novice/young teachers during the PhD programme? If so describe the system. |  |  |
|  | Is there a mentorship programme in place for PhD fellows/young teachers? If so, describe. |  |  |
|  |  |  |  |
| 2.3.9 | ***Student Networks and Representation*** |  |  |
|  | Are there any official networks or associations for PhD fellows? |  |  |
|  | What procedures does the university have for PhD fellow representation in established structures, boards, committee’s and other bodies? |  |  |
|  | What procedures does the university have in place for dealing with student complaints & appeals? |  |  |
|  |  |  |  |
| 2.3.10 | ***PhD Newsletter or Bulletin*** |  |  |
|  | Is there a PhD Bulletin at the university, if so describe purpose, content and frequency of publication |  |  |
|  |  |  |  |
|  |  |  |  |
| 3 | **Post Graduate Programmes** |  |  |
|  | Use the same framework for postgraduate programmes as used above on PhD programmes, but adjust where necessary. |  |  |
|  |  |  |  |
