# Supplementary material for: Needs assessment to strengthen capacity in water and sanitation research in Africa: experiences of the African SNOWS consortium
Source: Health Res Policy Syst. 2014 Dec 15;12:68. doi: 10.1186/1478-4505-12-68 (PMC4274706; doi:10.1186/1478-4505-12-68)
Supplement: Supplementary file 1 — Additional file 1: Zip file containing questionnaires used in the needs assessment. (ZIP 338 KB) [file 12961_2014_366_MOESM1_ESM.zip › SNOWS-Mapping.Template for Mapping Report-revised version.September.doc]

Mapping Report

University of xxx

Dated: xxx

Collated and presented by the Water and Sanitation group:

*Names of the Water & Sanitation group*

Introductory note by the Water and Sanitation group at the university: xxx (*e.g. on when formed, number of meetings held, etc.)*

1. **Research Management**
   1.1. **Organisation**

*[Provide the organisational chart/ structure of your university and describe which parts of the organisation are involved in research management within water, sanitation and environmental health – to the best of your knowledge if formal mapping is not viable.*

*Describe how the financial management of externally funded research programmes is organised at your institution (relevant to e.g. a next phase of SNOWS). ]*

*Describe by which means your university maintains and develops research management capacity (e.g. research management courses offered to staff)*

1.2. **Resources for SNOWS**

*[Describe the group you have mobilised for SNOWS, i.e. the SNOWS action group and resource persons (this group corresponds to the names you have included in the new template for SNOWS action group and resource persons- Annex 2-A).*

*You should qualify this description with an overview of current research projects of relevance to SNOWS (Annex 2-B).*

*Note the number of peer reviewed publications of relevance to SNOWS produced by the SNOWS action group 2005-10 (include the full list in Annex 2-C)*

*Describe whether the SNOWS action group and research persons have access to external resources complementary to those provided through the SNOWS project and which could be pooled towards common action/mutual benefits.*

*Provide information on sentinel sites which the SNOWS action group and resource persons use/have access to.]*

2. **PhD Programme**
   2.1. **Organisation**

*[Describe how the PhD programme is organised at your university.*

*If there is NO formal structure, e.g. no PhD programme but you actually have PhD students, then explain the informal set-up for enrolling and supporting PhDs at your university, and give priority to describing areas which the Water and Sanitation group at your university identifies as potential SNOWS action for enhancing the PhD organisation.*

*If the PhD programme is formalised describe the overall organisation briefly. Give priority to explaining those areas in more detail that the Water and Sanitation Group at your university identifies as areas that may be addressed by SNOWS action, e.g. revising policies; PhD rules & regulations; fundraising; monitoring & evaluation etc.*

*As a minimum the description should provide information on the requirements for PhDs to complete their doctorate degree. (If this information is available in an updated PhD handbook/rules and regulations document you may refer to this document but then remember to attach it to the report or provide direct URL.]*

2.2. **Training & Research environment / infrastructure**

*[Describe your research environment for PhD students. This description should include whether there are any taught courses for PhD students and whether such courses are mandatory or not. The description should also include information on your framework for providing quality supervision, access to research infrastructure including labs, IT, libraries and other support infrastructure for PhDs.*

*Describe by which means your university maintains and develops research supervisory skills, tutorial skills etc (e.g. PhD supervisor courses offered to staff).*

*Provide detailed description of those areas your Water and Sanitation group may wish to develop through SNOWS action.*

3. **Masters Programme**

*[This part should only be described if your university does not have PhD programmes. And would only cover the research part of the Masters programme, i.e. the thesis/dissertation part and research methodology courses of the Masters degree]*

*Describe the requirements for the Master thesis/dissertation. The description should also include information on research methodology courses and your framework for providing quality supervision, access to research infrastructure including labs, IT, libraries and other support infrastructure for Masters during their theses work.*

**Annexes**

| Annex 1 | SNOWS action group and resource persons |
| --- | --- |
| Annex 2-A | SNOWS-relevant research projects |
| Annex 2-B | Peer reviewed publications relevant to SNOWS |
| Annex 3 | List of Core Documents |
| Annex 4 | CV – scientific staff profile |
| Annex 5 | CV – PhD student profile |
