# Supplementary material for: Needs assessment to strengthen capacity in water and sanitation research in Africa: experiences of the African SNOWS consortium
Source: Health Res Policy Syst. 2014 Dec 15;12:68. doi: 10.1186/1478-4505-12-68 (PMC4274706; doi:10.1186/1478-4505-12-68)
Supplement: Supplementary file 1 — Additional file 1: Zip file containing questionnaires used in the needs assessment. (ZIP 338 KB) [file 12961_2014_366_MOESM1_ESM.zip › SNOWS-Template for Mapping Report.doc]

Mapping Report

University of xxx

Dated: xxx

Collated and presented by the Water and Sanitation group:

Names…

Introductory note by the Water and Sanitation group:	xxx

1. **Research Management**
   1.1. **Organisation**
        1.1.1. ***Organisational structure***
        1.1.2. ***Policies and strategies***
        1.1.3. ***Funding of Research***
        1.1.4. ***Financial Management***
   1.2. **Resources**
        1.2.1. ***Departments of relevance to SNOWS***
        1.2.2. ***Staff strength***
        1.2.3. ***Continued professional development and staff mobility***
        1.2.4. ***The Research Infrastructure***
        1.2.5. ***Study Sites and External Networks***
2. **PhD Programme**
   2.1. **Organisation**
        2.1.1. ***Organisational structure***
        2.1.2. ***Policies and strategies***
        2.1.3. ***Rules and Regulations for the PhD programme***
        2.1.4. ***Funding of PhD Research***
        2.1.5. ***Monitoring and evaluation***
   2.2. **Training & Research environment / infrastructure**
        2.2.1. ***Training – development of research and other skills***
        2.2.2. ***Supervision of PhDs***
        2.2.3. ***Technical support staff/lab technicians / librarians /IT support***
        2.2.4. ***Administrative Staff Strength***
   2.3. **PhD fellows**
        2.3.1. ***PhD Fellows in areas relevant to SNOWS***
        2.3.2. ***Mobility of PhD fellows***
        2.3.3. ***PhD admission***
        2.3.4. ***Monitoring progress***
        2.3.5. ***Research Budget***
        2.3.6. ***Conflict resolution***
        2.3.7. ***Assessment of the PhD fellow / dissertation and defence***
        2.3.8. ***Induction / support for young lecturers (PhD fellows required to teach/lecture)***
        2.3.9. ***Student Networks and Representation***
        2.3.10. ***PhD Newsletter or Bulletin***
3. **Post Graduate Programmes**
   3.1. **Organisation**
        3.1.1. ***Organisational structure***
        3.1.2. ***Policies and strategies***
        3.1.3. ***Rules and Regulations for the Post Graduate programmes***
        3.1.4. ***Funding of post graduate research***
        3.1.5. ***Monitoring and evaluation***
   3.2. **Training & Research environment / infrastructure**
        3.2.1. ***Training – development of research and other skills***
        3.2.2. ***Tutoring of post graduate students***
        3.2.3. ***Technical support staff/lab technicians / librarians /IT support***
        3.2.4. ***Administrative Staff Strength***
   3.3. **Post Graduate students**
        3.3.1. ***Post Graduate students in areas relevant to SNOWS***
        3.3.2. ***Mobility of Post Graduate students***
        3.3.3. ***Admission of Post Graduate students***
        3.3.4. ***Monitoring progress***
        3.3.5. ***Research Budget***
        3.3.6. ***Conflict resolution***
        3.3.7. ***Assessment of the post graduate students / thesis***
        3.3.8. ***Student Networks and Representation***
        3.3.9. ***Newsletter or Bulletin for Post Graduate students***
