# Supplementary material for: Needs assessment to strengthen capacity in water and sanitation research in Africa: experiences of the African SNOWS consortium
Source: Health Res Policy Syst. 2014 Dec 15;12:68. doi: 10.1186/1478-4505-12-68 (PMC4274706; doi:10.1186/1478-4505-12-68)
Supplement: Supplementary file 1 — Additional file 1: Zip file containing questionnaires used in the needs assessment. (ZIP 338 KB) [file 12961_2014_366_MOESM1_ESM.zip › Quest. ADMIN.rev.1.24.02.2010.doc]

SNOWS – Questionnaire for: *Adminstrative staff related to research management and management of post graduate and docratate programmes*

*Please, circle the correct code or fill in the response*

| **A. BACKGROUND INFORMATION** | |
| --- | --- |
| 1 | Questionnaire Serial Number: *[To be provided by local Team Coordinator]* |
| 2 | Name of SNOWS partner university:   1. Egerton University, Kenya 2. Kwame Nkrumah University of Science & Technology, Ghana 3. Mbara University of Science & Technology, Uganda 4. Tshwane University of Technology, South Africa 5. University of Gezira, Sudan 6. University of Venda, South Africa |
| 3 | Date of filling the questionnaire (dd/mm/yyyy): |
| **B. PERSONAL DATA** | |
| 5 | Category of respondent:   1. Administrative officer 2. Accountant 3. Head of Office: ……. 4. Other: …………… |
| 6 | Sex:   1. Male 2. Female |
| 7 | Age (in years): |
| 8 | 1. Formal education:…………..Year of gradutation:……….. 2. Additional education & courses attained prior to current position: ………………. 3. Additional education & courses attained during current assignment: ………………. |
| **C. FUNCTIONS** | |
|  | **Functions undertaken by you: (you can tic as many as required)**   1. Research management (other than PhD & post doc research) 2. Management of PhD programme 3. Management of Post Graduate programme 4. Fundraising 5. Project and programme administration 6. Project and programme accounting 7. Internal audit 8. Other, specify:………. |
|  | **Research management (other than PhD & post doc research) but only if ticked above** |
|  | List your key activities related to ***Research Management***  1. ………………  2. ………………  3. ……………… |
|  | Monitoring and reporting 1  Data for monitoring and reporting are readily available in the form required for you to deliver up to date and valid reports?  1. I fully agree  2. I agree to a large extent  3. I agree that this is mostly the case  4. I partly disagree  5. I disagree |
|  | Monitoring and reporting 2  Project and program progress reporting and completion reports are delivered on time.  1. I fully agree  2. I agree to a large extent  3. I agree that this is mostly the case  4. I partly disagree  5. I disagree |
|  | Monitoring and reporting 3  Project and program progress reporting and completion reports are of high quality.  1. I fully agree  2. I agree to a large extent  3. I agree that this is mostly the case  4. I partly disagree  5. I disagree |
|  | Improving research management 1  You believe that you have ideas that could significantly contribute to improving effectiveness of research management at your university.  1. I fully agree  2. I agree to a large extent  3. I agree that this is mostly the case  4. I partly disagree  5. I disagree |
|  | Improving research management 2  You are involved in sharing ideas for improving the effectiveness of research management (eg. by being involved in internal evaluations or reviews of research management?)  1. I fully agree  2. I agree to a large extent  3. I agree that this is mostly the case  4. I partly disagree  5. I disagree |
|  | **Management of PhD programmes - but only if ticked above** |
|  | List your key activities related to ***Management of PhD programmes***  1. ………………  2. ………………  3. ……………… |
|  | Registration and enrolment of PhDs  Regristration and enrolment procedures are effective  1. I fully agree  2. I agree to a large extent  3. I agree that this is mostly the case  4. I partly disagree  5. I disagree |
|  | Monitoring and Reporting Progress of PhDs 1  Data for monitoring and reporting on PhD progress are readily available in the form required for you to deliver up to date and valid reports?  1. I fully agree  2. I agree to a large extent  3. I agree that this is mostly the case  4. I partly disagree  5. I disagree |
|  | Monitoring and Reporting Progress of PhDs 2  PhD progress reports are delivered on time.  1. I fully agree  2. I agree to a large extent  3. I agree that this is mostly the case  4. I partly disagree  5. I disagree |
|  | Monitoring and Reporting Progress of PhDs 3  PhD progress reports are made available to all relevant stakeholders.  .  1. I fully agree  2. I agree to a large extent  3. I agree that this is mostly the case  4. I partly disagree  5. I disagree |
|  | Monitoring and Reporting Progress of PhDs 4  The PhD register/ information systems is up-to date and reports reliable  .  1. I fully agree  2. I agree to a large extent  3. I agree that this is mostly the case  4. I partly disagree  5. I disagree |
|  | Monitoring and Reporting Progress of PhDs 5  The PhD register/ information system / online tracking system is generally effective and reliable  .  1. I fully agree  2. I agree to a large extent  3. I agree that this is mostly the case  4. I partly disagree  5. I disagree |
|  | Rules and regulations for PhDs 1  How knowledgable do you find yourself on the PhD rules and regulations   1. Very knowledgeable 2. Moderately knowledgeable 3. Non-knowledgeable; specify why:…………….. 4. I don’t know |
|  | Rules and regulations for PhDs 2  The current rules and regulations cover all relevant aspects of the PhD programme  1. I fully agree  2. I agree to a large extent  3. I agree that this is mostly the case  4. I partly disagree  5. I disagree |
|  | Rules and regulations for PhDs 3 |
|  | Information required for your contribution to the effective adminstration of the PhD programme  I have easy access to the relevant information that I need to perform well.  1. I fully agree  2. I agree to a large extent  3. I agree that this is mostly the case  4. I partly disagree  5. I disagree |
|  | Improving Management of PhD programmes 1  You believe that you have ideas that could significantly contribute to improving the management of PhD programmes at your university.  1. I fully agree  2. I agree to a large extent  3. I agree that this is mostly the case  4. I partly disagree  5. I disagree |
|  | Improving Management of PhD programmes 2  You are involved in sharing ideas for improving the effectiveness of PhD programmes (eg. by being involved in internal evaluations or reviews of PhD administration & management?)  1. I fully agree  2. I agree to a large extent  3. I agree that this is mostly the case  4. I partly disagree  5. I disagree |
|  |  |
|  | **And so forth for :**  -Management of Post Graduate programme  -Fundraising  -Project and programme administration  -Project and programme accounting  -Internal audit |
| **D. CONTINUOUS PROFESSIONAL DEVELOPMENT** | |
|  | You have good opportunities for professional development and to be upgraded on relevant skills  1. I fully agree  2. I agree to a large extent  3. I agree that this is mostly the case  4. I partly disagree  5. I disagree |
|  | When was the last time you attended courses aimed at your professional development?  1. 2009-10  2. 2006-8  3. 2005 or before  4. never |
|  | I find that I could be more effective in my daily work if I attended courses in continuous professional development  1. I fully agree  2. I agree to a large extent  3. I agree that this is mostly the case  4. I partly disagree  5. I disagree |
|  | Provide list of areas that you consider of importance for your continuous professional development  1………….  2………….  3…………. |
